# Chemical Investigation of the Indonesian Tunicate *Polycarpa aurata* and Evaluation of the Effects Against *Schistosoma mansoni* of the Novel Alkaloids Polyaurines A and B

**DOI:** 10.3390/md17050278

**Published:** 2019-05-10

**Authors:** Marcello Casertano, Concetta Imperatore, Paolo Luciano, Anna Aiello, Masteria Yunovilsa Putra, Roberto Gimmelli, Giovina Ruberti, Marialuisa Menna

**Affiliations:** 1 The NeaNat Group, Department of Pharmacy, University of Naples “Federico II”, Via D. Montesano 49, 80131 Napoli, Italy; marcello.casertano@unina.it (M.C.); cimperat@unina.it (C.I.); pluciano@unina.it (P.L.); aiello@unina.it (A.A.); 2 Research Center for Oceanography, Indonesian Institute of Sciences, Jl Pasir Putih Raya 1, DKI Jakarta 14430, Indonesia; mast001@lipi.go.id; 3 Institute of Cell Biology and Neurobiology, National Research Council, Campus A. Buzzati-Traverso, Via E. Ramarini, 32, 00015 Monterotondo (Roma), Italy; roberto.gimmelli@ibcn.cnr.it (R.G.); giovina.ruberti@cnr.it (G.R.)

**Keywords:** natural products, ascidians, *Polycarpa aurata*, 1,2,4-thiadiazole alkaloids, *Schistoma mansoni*

## Abstract

A deep study of the metabolic content of the tunicate *Polycarpa aurata*, collected from Indonesian coast, afforded the isolation of two novel alkaloids, polyaurines A (**1**) and B (**2**), along with two new *p*-substituted benzoyl derivatives (**3** and **4**) and four known compounds (**5**–**8**). The structural elucidation of the new secondary metabolites was assigned by 1D, 2D NMR, and HRESIMS techniques. Computational studies resulted a useful tool to unambiguously determine in polyaurine B the presence of rarely found 1,2,4-thiadiazole ring. The effects of polyaurines A and B on mammalian cells growth and on the viability of different blood-dwelling *Schistosoma mansoni* (phylum: Platyhelminthes) stages, as well as egg production, were evaluated. Both compounds resulted not cytotoxic; interestingly some of the eggs produced by polyaurine A-treated adult pairs in vitro are smaller, deformed, and/or fragmented; therefore, polyaurine A could represent an interesting bioactive natural molecule to be further investigated.

## 1. Introduction

The solitary ascidian *Polycarpa aurata* (Quoi and Gaimard, 1834), a common constituent of the benthic invertebrate community of Indo-Pacific coral reefs has proved to be an extremely rich source of chemo diversity, especially of unique alkaloid-type structures. Typically, the heterocycle parts commonly found in the structures of natural products contain one or two heteroatoms, most frequently nitrogen and/or oxygen and occasionally sulfur [1]. Indeed, many alkaloids with rare sulfur-containing functional groups and/or featuring different heterocycle portion have been isolated from this marine invertebrate. Examples are the dimeric disulfide alkaloid polycarpine and its derivatives [2], the polycarpamines A–E [3], the 2-aminoimidazole polycarpaurines A–C [4], the indole alkaloid *N*,*N*-didesmethylgrossularine-1 [2], and the unique 1,2,4-thiadiazole alkaloids polycarpathiamines A and B [5]. In addition to these very unusual alkaloids, a series of both nitrogen-containing and non-nitrogenous benzoyl derivatives were also isolated from this ascidian species [6]. Most of these compounds exhibited various biological activities, such as antifungal [3], cytotoxic [2,4,7,8], pro-apoptotic [9], and inosine monophosphate dehydrogenase (IMPDH) inhibiting activities [2]. The extremely high chemical diversity of *P. aurata* and observation that different collections of this organism yielded different metabolites patterns suggested that the ascidian may not be the real producer of these unique compounds and that their bioproduction could involve associated microorganisms or the planktonic biomass [3].

**Figure 1:**
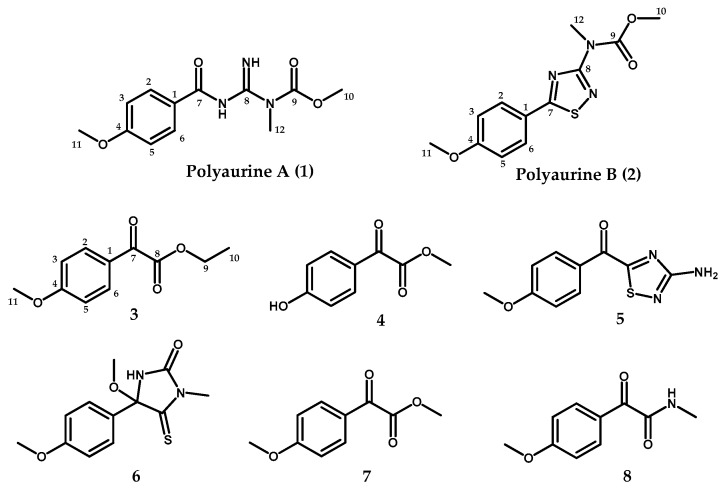
Structures of compounds **1**–**8**.

In the frame of our continuing search for novel bioactive compounds from marine ascidians, we have investigated a sample of *P. aurata* collected along the coast of Siladen (Indonesia); the chemical analysis of the methanol extract of this organism afforded four new metabolites (compounds **1**–**4**, Figure 1), along with four known compounds (**5**–**8**, Figure 1) previously reported from *P. aurata* and other *Polycarpa* species. Compounds **5**–**8** were identified as polycarpathiamine B (**5**) [5], 4-methoxy-4-(4-methoxyphenyl)-1-methyl-5-thioxoimidazolidin-2-one (**6**) [2,7], methyl 2-(4-methoxyphenyl)-2-oxoacetate (**7**) [6], and 2-(4-methoxyphenyl)-*N*-methyl-2-oxoacetamide (**8**) [6] by comparison of their spectral data with those reported in the literature. The structures of the two new benzoyl derivatives ethyl 2-(4-methoxyphenyl)-2-oxoacetate (**3**) and methyl 2-(4-hydroxyphenyl)-2-oxoacetate (**4**) were also easily assigned based on their spectral properties compared to those of the strictly related known compounds **7** and **8** [6].

We describe herein the isolation and structure elucidation of the two novel alkaloids **1** and **2** that we named polyaurines A and B, respectively. Notably, the structure of polyaurine B contains the “heteroatom-rich” 1,2,4-thiadiazole nucleus, which is really uncommon in natural products [5]; compound **2** represents, to our knowledge, the first example of a 3-(*N-*methyl-methylcarbamate) substituted 1,2,4-thiadiazole alkaloid.

Despite the small amounts of compounds that we succeeded in obtaining from the extraction of the ascidian sample, we have investigated the effects of the two novel alkaloids, **1** and **2**, on the viability of *Schistosoma mansoni* larval and adult stages and egg production. Schistosomiasis is one of the most important parasitic diseases, with more than 200 million people infected globally by the Platyhelminthes of the genus Schistosoma. The parasite has a complex life cycle that includes several morphological phenotypes in the intermediate fresh-water snail host (*Biomphalaria* spp.) and in the mammalian definitive host. Adult *S. mansoni* worms live predominantly in the small inferior mesenteric blood vessels where the mated females release hundreds of eggs daily. Excretion of eggs within the fecal material maintains the parasites life-cycle. The eggs trapped in the liver evoke inflammation and host-immune reactions leading to the formation of granuloma around the eggs and progressive organ damage and pathology [10,11]. Here, we show that some of the eggs produced by polyaurine A-treated adult pairs in vitro are smaller, deformed, and/or fragmented. Importantly, the polyaurine A (**1**) is not cytotoxic against mammalian cells; therefore, it could represent an interesting bioactive natural molecule to be further investigated.

## 2. Results

### 2.1. Isolation and Structure Elucidation of Compounds ***1**–**2***

Several fresh specimens of *P. aurata* were extracted with methanol and, then, with chloroform. Solvent partitioning of the combined extracts yielded a lipid soluble portion, which was fractionated by silica gel medium pressure chromatography. TLC and ^1^H NMR guided separations and purifications by HPLC of the obtained fractions afforded compounds **1**–**8** in the pure state.

The HRESIMS of polyaurine A (**1**) showed peaks at *m/z* 266.1129 [M + H]^+^ and 288.0948 [M + Na]^+^ suggesting the molecular formula C_12_H_16_N_3_O_4_ with seven degrees of unsaturation. The ^1^H and ^13^C NMR data obtained for **1** and assigned through 2D NMR experiments (Table 1) evidenced the presence of a *p*-methoxyphenyl unit. In particular, the deshielding of two aromatic protons at *δ*_H_ 8.17 (2H, d, *J* = 8.2 Hz, H-2/6) and *δ*_H_ 6.89 (2H, d, *J* = 8.2 Hz, H-3/5) suggested the presence of a *para* oxygenated benzoyl system. Four carbon resonances, two protonated (*δ*_C_ 131.1 (C-2 and C-6) and 113.0 (C-3 and C-5)) and two unprotonated (*δ*_C_ 162.4 (C4) and 130.7 (C-1)), were assigned by HSQC and HMBC experiments (Table 1), to this aromatic moiety. The presence of a methoxy-functionality was inferred by the proton and carbon resonances at *δ*_H_ 3.84 (3H, s, Me-11) and *δ*_C_ 55.2 (C-11), respectively. HMBC correlations between these methoxyl protons and both the aromatic quaternary carbon at *δ*_C_ 162.4 (C-4) and the carbons at *δ*_C_ 113.0 (C-3/5) allowed to link this group at C-4. A 4-methoxybenzoyl unit was thus evident from the fragment peak at *m/z =* 135 in the MS/MS spectrum of **1** and from the HMBC correlation of the aromatic proton signals at *δ*_H_ 8.17 (H-2/6) with the carbonyl resonance at *δ*_C_ 177.9. This unit accounted for five of the seven degrees of unsaturation suggested by the molecular formula of **1**.

**Figure 2:**
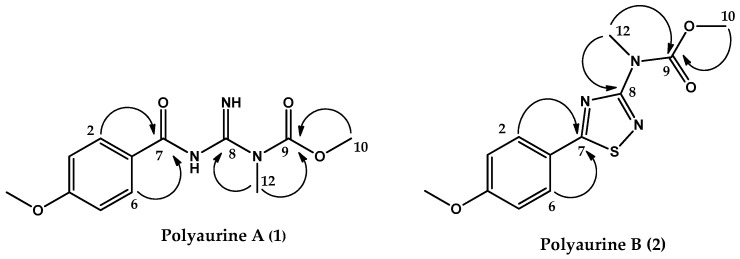
Key HMBC correlations of compounds **1** and **2**.

Additional features of the proton spectrum of **1** were two downfield signals at *δ*_H_ 9.28 (1H, br.s, *N*H) and *δ*_H_ 10.58 (1H, br.s, *N*H), as well as two methyl singlets at *δ*_H_ 3.86 (3H, s, Me-10) and 3.52 (3H, s, Me-12). The methyl signals could be assigned, based on their chemical shift values and both HSQC and HMBC correlations, to a nitrogen linked methyl group (*δ*_C_ = 32.3) and to a methoxyl group (*δ*_C_ 53.8), respectively. Two unprotonated sp^2^ carbon resonances remained in the ^13^C NMR spectrum of **1**, at *δ*_C_ 159.8 and 156.7. According to the molecular formula of **1** and based on the whole series of the HMBC correlations (Figure 2), a methyl-guanidine unit and a carbamate function were identified. Key correlations were those between the *N*-linked methyl group (Me-12, *δ*_H_ 3.52) and both the sp^2^ carbon resonance at *δ*_C_ 159.8 and 156.7, the latter being correlated to the methoxyl group resonating at *δ*_H_ 3.86 (Me-10). Therefore, compound (**1**) was identified as depicted in Figure 2 and was named polyaurine A.

**Figure 3:**
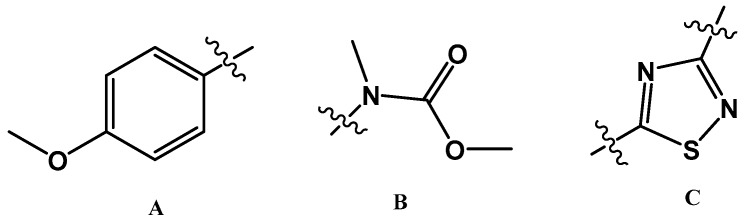
Subunits A–C of the structure of **2**.

Polyaurine B (**2**) had the molecular formula C_12_H_13_N_3_O_3_S, as deduced from the HRESIMS spectrum (positive ion mode), which showed pseudomolecular ion peaks at *m/z* 280.0741 [M + H]^+^ and *m/z* 302.0558 [M + Na]^+^. The molecular formula indicated eight degrees of unsaturation, one more than polyaurine A. An extensive NMR analysis conducted on the molecule allowed the initial identification of three subunits in the structure (subunits A–C, Figure 3) that were subsequently connected through further HMBC experiments.

The presence of the *p*-methoxyphenyl group (subunit A, Figure 3), which also appears in the structure of polyaurine A (**1**), was deduced from the aromatic methine resonances present in the ^1^H and^13^C NMR spectra (CDCl_3_) [*δ*_H_ 7.88 (2H, d, *J* = 7.9 Hz, H-2/6); *δ*_H_ 6.96 (2H, d, *J* = 7.9 Hz, H-3/5); *δ*_C_ 128.9 (C-2 and C-6), *δ*_C_ 114.7 (C-3 and C-5)], the methoxyl signal at *δ*_H_ 3.87 (Me-11), and the unprotonated carbon signals at *δ*_C_ 162.7 (C4), 123.2 (C-1), and 55.5 (C-11), which were assigned to this aromatic system on the basis of two-dimensional HSQC and HMBC experiments (see Table 1). The latter experiment also showed that the methoxyl protons at *δ*_H_ 3.87 were correlated with both the quaternary carbon signal at *δ*_C_ 162.7 (C-4) and the methine signal at *δ*_C_ 114.7 (C-3/5). The remaining signals in the ^1^H NMR spectrum of **2** were two additional methyl singlets at *δ*_H_ 3.54 (3H, Me-12) and 3.85 (3H, Me-10); the relevant carbons, identified by the HSQC experiment, were at *δ_C_* 36.1 and 53.6, respectively. These proton and carbon resonances were reasonably ascribed to an *N*-linked methyl and a methoxyl group, respectively. Both singlets at *δ*_H_ 3.54 and 3.85 were correlated in the HMBC spectrum to the quaternary *sp^2^* carbon resonance at *δ*c 154.9. Based on these data, the *N*-methyl-methylcarbamate functionality (subunit B, Figure 3) was identified in **2**.

According to the molecular formula assigned to **2** by mass spectrometric analysis, one sulfur, two carbon, and two nitrogen atoms still remained to be placed in the molecule; moreover, three degrees of unsaturation had to be satisfied and only two quaternary *sp^2^* carbon signals, at *δ*c 187.1 and 165.8, remained to be assigned. Thus, we hypothesized the third structural subunit in the molecule of compound **2** to be a 1,2,4-thiadiazole ring (subunit C, Figure 3), the only thiadiazole regioisomer known to occur naturally [1] and already found in the structure of polycarpathiamines previously discovered from *P. aurata* [5]. Comparison of the chemical shift values of compound **2**, and precisely evaluation at *δ*c values of the two carbons assigned to the heterocyclic ring, with those reported in the literature for both natural [5,12,13,14] and synthetic 1,2,4-thiadiazole alkaloids [14,15,16,17] allowed to substantiate this assumption.

The three identified structural subunits A–C were finally connected on the basis of key correlations observed in the HMBC spectrum. In detail, a correlation was observed between the aromatic proton signal at *δ*_H_ 7.88 (H-2/6) and the carbon signal at *δ*c 187.1, relative to the carbon between the atoms of nitrogen and sulfur of the thiadiazole ring (C-7), whereas the protons of the *N*-linked group of subunit B (*δ*_H_ 3.54) showed a correlation with the remaining carbon of the subunit C resonating at *δ*c 165.8. All these data allowed to propose the structure shown in Figure 2 for compound **2**, which is named polyaurine B.

The first reported example of a natural product containing the 1,2,4-thiadiazole heterocyclic moiety has been the alkaloid dendrodoine, isolated from the marine ascidian *Dendrodoa grossularia* (Styelideae) [12]. This compound has been the only example until 2012, when a pair of enantiomeric indole alkaloids containing the 1,2,4-thiadiazole unit were isolated from the plant *Isatis indigotica* [13]. The 3-amino substituted 1,2,4-thiadiazole alkaloids polycarpathiamines have been isolated in 2013 from a marine ascidian [5], and, recently, the 1,2,4-thiadiazole alkaloid penicilliumthiamine B has been isolated from the fungus *Penicillium oxalicum* [14].

### 2.2. Validation of the Polyaurine B (***2***) Structure by Theoretical QM Calculations

To unequivocally prove the existence of the unusual 1,2,4-thiadiazole heterocyclic ring, structure elucidation of these compounds has been confirmed by synthesis [14,15,16,17] with respect of many spectroscopic evidences as well as biogenetic considerations. Instead, we obtained further support to the methyl 5-(4-methoxyphenyl)-1,2,4-thiadiazol-3-yl)(methyl)carbamate structure proposed for polyaurine B, through the quantum mechanical calculation of its ^13^C NMR chemical shifts profile and application of DP4+ statistical analysis. This approach, relying on the use of QM calculated NMR parameters profiles combined with refined data processed by appropriate “computational toolboxes,” is becoming increasingly important as a valuable theoretical supplement to the experimental spectroscopic, both chiroptical and NMR, data in structure assignment of natural products [15].

**Figure 4:**
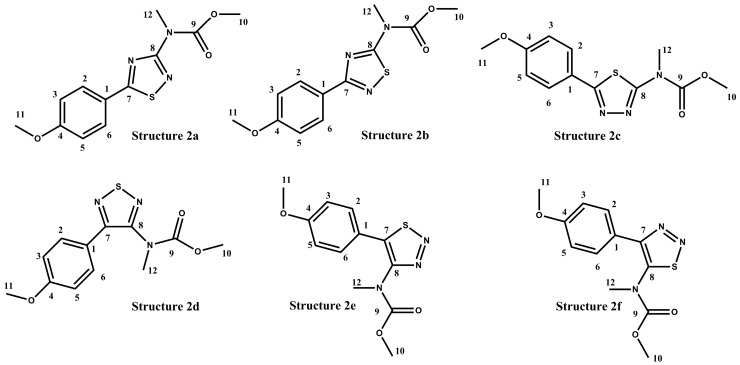
Isomeric alternative structures considered for polyaurine B (**2**).

In the case of polyaurine B (**2**), in addition to the structure proposed on the basis of the obtained experimental data and literature reports (**2**, Figure 1), six possible alternative isomeric structures (**2a**–**2f,** Figure 4) were considered, quite compatible with the experimental NMR data.

For these six structures, the ^13^C NMR chemical shift values were calculated using the GIAO method with the DFT technique. For this purpose, all the structures were previously subjected to an optimization of the geometry and energy optimization using DFT with the mPW1PW91/6-311+G(2d,p) functional and basis set combination [18,19,20,21,22]. The theoretical ^13^C NMR chemical shift values predicted for **2a**–**2f** are reported in Table 2, in comparison to the experimental data obtained for the natural metabolite.

The analysis of the theoretical ^13^C NMR data showed that the structure **2a** was the correct isomer. The ^13^C MAE (mean absolute errors) of 3.2 ppm and ^13^C CMAE of 1.7 ppm (correct mean absolute errors) for the structure **2a** was below 5 ppm in the ^13^C NMR data, which is considered within the range of an acceptable DFT-NMR calculation for theoretical values. The theoretical ^13^C NMR data was also analyzed through the DP4+ statistical analysis, which can be used to distinguish between constitutional isomers. The DP4+ analysis (Table 3) showed that ^13^C NMR data were most consistent with the **2a** structure (100% probability).

### 2.3. Biological Activities of Compounds ***1*** and ***2***

Polyaurines A (**1**) and B (**2**) were tested for their effects on the viability of NIH-3T3 mammalian cells and the *Schistosoma mansoni* parasite. Both compounds were not active and showed, in a dose-curve response, an IC_50_ higher than 100 μM against both mammalian cells and larval stage (schistosomula) of *Schistosoma mansoni*.

**Figure 5:**
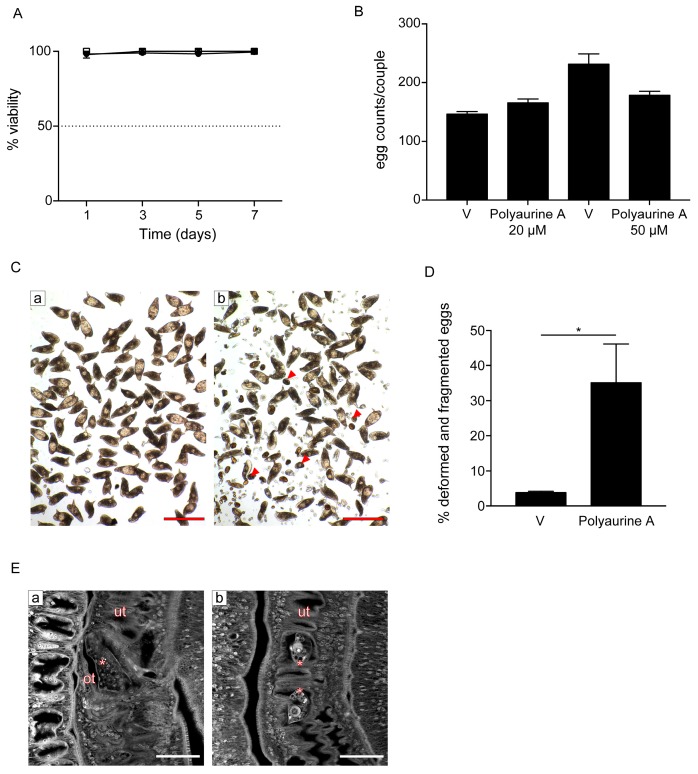
Effects of polyaurine A on *S. mansoni* adult parasites. (**A**) Adult pairs viability assays. Worm pairs were incubated with vehicle (DMSO) (circle), polyaurine A (**1**) 20 μM (square), or 50 μM (triangle), and viability is shown as the percentage of vehicle-treated samples indicated as 100%. The data shown represent the mean of three independent experiments ± SEM. (**B**) Worm pairs were incubated with vehicle (V) or polyaurine A (**1**) at the indicated concentrations, and eggs were counted at 72 h. Egg counts were normalized to the number of worm couples. The data shown represent the mean of three independent experiments ± SEM. (**C**) Representative microscopy images of *S. mansoni* eggs laid in vitro by worm pairs treated with vehicle (a) or 50 μM of polyaurine A (**1**) (b). Red arrows indicate the deformed or fragmented eggs (b). Scale bars = 200 μm. (**D**) Histograms represent the percentage of deformed and/or fragmented eggs counted at 72 h. Approximately 500 eggs were counted in 3 independent experiments (150–200 eggs/experiment). Asterisk in the figure indicates significant *t*-test *p*-value (* *p* < 0.05) relative to the comparison of 50 μM of polyaurine A-treated samples with the vehicle V-treated ones. (**E**) Representative confocal scanning laser images of adult *S. mansoni* pairs treated for 72 h with vehicle or 50 μM of polyaurine A. Eggs in the ootype (a, vehicle) or uterus (b, polyaurine A) are indicated by asterisks. Abbreviations: ot, ootype; ut, uterus. Scale-bars = 50 μm. In all experiments, vehicle-treated parasites or eggs received the same amount of DMSO (in volume) as the polyaurine A (**1**)-treated ones.

The activity of polyaurine A was further investigated on adult *S. mansoni* pairs (Figure 5) with respect to polyaurine B considering the low available quantity for the latter.

Interestingly, while it did not impact parasite viability at both 20 and 50 μM during the seven days of observation (Figure 5A), it impaired egg production in vitro (Figure 5B–D). The total number of eggs laid by females upon treatment of *S. mansoni* pairs with both 20 and 50 μM of polyaurine A (**1**) or vehicle (DMSO) was similar (Figure 5B). However, some eggs laid in vitro by polyaurine A-treated parasites appeared deformed and several fragments of eggs were present in the plate dish, along with sperms and vitelline cells (Figure 5C). The number of deformed/fragmented eggs was quantified, and it resulted to be increased in the polyaurine A-treated samples in comparison to the DMSO-treated ones (*p* < 0.05) (Figure 5D). By carmine red-staining and confocal microscopy analyses, smaller size eggs were observed in the ootype and/or uterus of polyaurine A-treated parasites (Figure 5E). The eggs are crucial players in schistosomiasis for the maintenance of the parasite life-cycle, the definitive host tissue damage, and the development of the disease. Overall, these results indicate that polyaurine A is not cytotoxic on mammalian cells, but it is active on parasite egg production. Therefore, polyaurine A is an interesting bioactive natural molecule to be further investigated.

## 3. Materials and Methods

### 3.1. General Experimental Procedures

HRESIMS (positive mode) was performed on a Thermo LTQ Orbitrap XL mass spectrometer (Thermo-Fisher, San Josè, CA, USA). The spectra were recorded by infusion into the ESI source using MeOH as solvent. ^1^H NMR (700 MHz and 500 MHz) and ^13^C NMR (175 MHz and 125 MHz) spectra were recorded with an Agilent INOVA spectrometer (Agilent Technology, Cernusco sul Naviglio, Italy); chemical shifts were referenced to the residual solvent signal (CDCl_3_: δ_H_ = 7.26, δ_C_ = 77.0). Homonuclear ^1^H connectivities were determined by COSY experiments. Two and three bond ^1^H-^13^C connectivities were determined by gradient 2D HMBC experiments optimized for a ^2,3^*J* of 8 Hz. High performance liquid chromatography (HPLC) separation was achieved on a Knauer K-501 apparatus equipped with a Knauer K-2301 RI detector (LabService Analytica s.r.l., Anzola dell’Emilia, Italy).

### 3.2. Collection, Extraction, and Isolation

Specimens of *Polycarpa aurata* were collected along the coast of Siladen (Indonesia, 1°37′41″ N 124°48′01″ E) in the autumn of 2012. The identification of the organisms was carried out by Dr. Masteria Yunovilsa Putra. They were frozen immediately after collection and kept frozen until extraction. A voucher specimen is deposited at the Department of Pharmacy, University of Naples “Federico II”, Naples, Italy.

Fresh thawed animals (39.8 g dry weight after extraction) were homogenized and extracted with MeOH (3 × 400 mL) and then with CHCl_3_ (3 × 400 mL). Extracts were combined and concentrated in vacuo; the resulting aqueous residue was then partitioned giving EtOAc, n-BuOH, and aqueous extracts. The ethyl acetate-soluble material was chromatographed by MPLC over a silica gel column followed using an increasing gradient elution (100% *n*-hexane→ *n*-hexane:EtOAc 9:1→ *n*-hexane:EtOAc 7:3→*n*-hexane:EtOAc 1:1→*n*-hexane:EtOAc 3:7→*n*-hexane:EtOAc 1:9→ 100% EtOAc→ EtOAc:MeOH 9:1→100% MeOH→100% CHCl_3_) to yield eighteen fractions 1–18. The fractions (6–9) eluted with *n*-hexane:EtOAc 1:1(*v/v*) were further purified by HPLC. Fraction 6 was chromatographed by HPLC Luna 3 μm Silica column, *n*-hexane:EtOAc (95:5) and yielded in pure form polyaurine A (**1**, t*_R_* 9.2 min, 1.7 mg), polyaurine B (**2**, t*_R_* 12.7 min, 0.5 mg), compound **3** (t*_R_* 6.5 min, 0.2 mg), compound **4** (t*_R_* 19.1 min, 0.6 mg), together with a mixture of compound **6** and **7** (t*_R_* 20.6 min). HPLC on Luna 3 μm PFP column, MeOH:H_2_O (75:25), allowed to separate into individual compounds, the latter mixture yielding compound **6** (t*_R_* 8.3 min, 0.5 mg) and compound **7** (t*_R_* 6.2 min, 0.7mg) in the pure state. Fraction 7 was analyzed by HPLC on Luna 3 μm Silica column, *n*-hexane:EtOAc (85:15) afforded compound **8** (t*_R_* 10.7 min, 0.5 mg) in pure state. Fraction 8 was analyzed by HPLC on Luna 3 μm Silica column, *n*-hexane:EtOAc (9:1), yielding a fraction mainly composed of **5** (t*_R_* 22.4 min, 0.8 mg) which has been further purified by HPLC on a RP-18 column (Luna 3μm PFP), eluting with MeOH:H_2_O (7:3), thus affording compound **5** (t*_R_* 5.1 min, 0.4 mg) as pure compound.

Polyaurine A (**1**): yellow powder; ^1^H and ^13^C NMR data (CDCl_3_) are reported in Table 1; 2D NMR data, Figures S4–S6; HRMS (ESI): *m/z* 266.1129 [M + H]^+^ (calcd. for C_12_H_16_O_4_N_3_: 266.1135); *m/z* 288.0948 [M + Na]^+^ (calcd. for C_12_H_15_O_4_N_3_Na: 288.0955) (Figure S1).

Polyaurine B (**2**): yellow powder; ^1^H and ^13^C NMR data (CDCl_3_) are reported in Table 1; 2D NMR data, Figures S10–S12; HRMS (ESI): *m/z* 280.0741 [M + H]^+^ (calcd. for C_12_H_14_O_3_N_3_S: 280.0750); *m/z* 302.0558 [M + Na]^+^ (calcd. for C_12_H_13_O_3_N_3_SNa 302.0570) (Figure S7).

Compound **3**: white powder; ^1^H NMR (CDCl_3_) spectrum is reported in Supplementary Materials (Figure S14); HRMS (ESI): *m/z* 209.0810 [M + H]^+^ (calcd. for C_11_H_13_O_4_: 209.0808); *m/z* 231.0630 [M + Na]^+^ (calcd. for C_11_H_12_O_4_Na: 231.0628); *m/z* 231.0370 [M + K]^+^ (calcd. for C_11_H_12_O_4_K:247.0367) (Figure S13).

Compound **4**: white powder; ^1^H NMR (CDCl_3_) spectrum is reported in Supplementary Materials (Figure S16); HRMS (ESI): *m/z* 181.0491 [M + H]^+^ (calcd. for C_9_H_9_O_4_: 181.0495); *m/z* 203.0309 [M + Na]^+^ (calcd. for C_9_H_8_O_4_Na:203.0315) (Figure S15).

Compound **5**: white powder; ^1^H NMR (CDCl_3_) spectrum is reported in Supplementary Materials (Figure S18); HRMS (ESI): *m/z* 236.0505 [M + H]^+^ (calcd. for C_10_H_10_N_3_O_2_S: 236.0488); *m/z* 258.0327 [M + Na]^+^ (calcd. for C_10_H_9_N_3_O_2_SNa: 258.0308 (Figure S17).

Compound **6**: yellow powder; ${\left[\alpha \right]}_{D}^{25}$ +33.5 (*c* 0.0002, CH_3_OH); ^1^H NMR (CDCl_3_) spectrum is reported in Supplementary Materials (Figure S20); HRMS (ESI): *m/z* 289.0641 [M + Na]^+^ calcd. for C_12_H_14_N_2_O_3_SNa: 289.0617 (Figure S19).

Compound **7**: colourless oil; ^1^H NMR (CDCl_3_) spectrum is reported in Supplementary Materials (Figure S22); HRMS (ESI): *m/z* 195.0647 [M + H]^+^ (calcd. for C_10_H_11_O_4_: 195.0652); *m/z*: 217.0465 [M + Na]^+^ (calcd. for C_10_H_10_O_4_Na: 217.0471); *m/z* 233.0205 [M + K]^+^ (calcd. for C_10_H_10_O_4_K^+^: 233.0211) (Figure S21).

Compound **8**: colourless oil; ^1^H NMR (CDCl_3_) spectrum is reported in Supplementary Materials (Figure S24); HRMS (ESI): *m/z* 194.0806 [M + H]^+^ (calcd. for C_10_H_12_NO_3_: 194.0812); *m/z* 216.0625 [M + Na]^+^ (calcd. for C_10_H_11_NO_3_Na: 216.0631) (Figure S23).

### 3.3. Biological Activity

#### 3.3.1. Ethical Statement

Animal work was approved by the National Research Council, Institute of Cell Biology and Neurobiology animal welfare committee (OPBA) and by the competent authorities of the Italian Ministry of Health, DGSAF, Roma (authorizations no. 25/2014-PR and no. 336/2018-PR). All experiments were conducted in respect to the 3R rules according to the ethical and safety rules and guidelines for the use of animals in biomedical research provided by the relevant Italian law and European Union Directive (Italian Legislative Decree 26/2014 and 2010/63/EU) and the International Guiding Principles for Biomedical Research involving animals (Council for the International Organizations of Medical Sciences, Geneva, Switzerland).

#### 3.3.2. Maintenance of the *S. mansoni* Life-Cycle

A Puerto Rican strain of *S. mansoni* was maintained in albino *Biomphalaria glabrata*, as the intermediate host, and ICR (CD-1) outbred female mice as the definitive host as previously described [23]. Female 7- to 8-week-old mice (Envigo, Udine, Italy) were infected with 150–200 double sex *S. mansoni* cercariae by the tail immersion technique.

#### 3.3.3. Preparation of Parasites, Viability Assays, and Eggs Analysis

Schistosomula were prepared by mechanical transformation of cercariae, and adult worm pairs were isolated by reversed perfusion of the hepatic portal system and mesenteric veins of 7–8 weeks post-infection. The protocols for the parasite preparation have been previously reported [23]. The schistosomula ATP-based assay was performed in 96-wells/cell culture black microplate (Greiner Bio-One S.r.l, Roma, Italy, #655090) with the CellTiterGlo (CTG) (Promega, Madison, WI, USA) as previously described but using 150–200 schistosmula/well and 50 μL of CTG [23]. The luminescence signal was measured with a Varioskan Lux and the Skanit software (ThermoFisher Scientific, Waltham, MA, USA). The percentage of dead schistosomula for each compound was calculated as the ATP reduction against vehicle and gambogic acid (50 μM) used, respectively, as negative (0%) and positive control (100%). For the adult worm parasites assays, 5 adult male–female pairs were cultured in tissue culture medium. Vehicle (DMSO) or polyaurine A were added to the parasite at 20 and 50 μM concentrations only once 24 h upon parasite isolation from infected mice and observed for seven days. A survival score was assigned daily based on phenotype (plate-attached, movement, color, gut peristalsis, tegument damage, male–female pairing) under a MZ12 stereomicroscope (Leica Microsystems, Mannheim, Germany) as previously reported [23]. The percentage severity score (viability) was assigned in three independent experiments, relative to DMSO. For in vitro eggs laying, 5 worm adult pairs were incubated with DMSO or polyaurine A (**1**), the number of eggs was counted at 72 hours, and normalized to parasite pairs. Images of eggs were recorded with a BX41 microscope and a brightfield objective 10× served by a SPOT RT 220-3 Diagnostic Instrument Inc camera (Olympus, Waltham, MA, USA).

#### 3.3.4. Confocal Laser Scanning Microscopy Analysis

Carmine-red staining was performed on parasite pairs as previously described [24]. Images were taken on a FV1200 confocal laser-scanning microscope using an UPlanFLN 40× immersion oil objective (NA = 1.30) and a multiline argon laser at 488 nm as the excitation source (Olympus, Waltham, MA, USA). The images were collected as a single stack.

#### 3.3.5. Viability Mammalian Cell Assay

NIH-3T3 mouse embryonic fibroblasts, plated in 96-well plates at day 0 were treated with increasing concentrations of compound (0.4 × 10^4^ cells/well) **1** or **2** (from 0.390 up to 100 μM) in complete tissue culture medium and cultured for 72 h at 37 °C in 5% CO_2_. MTT was used at 1 mg/mL and formazan crystals were solubilized with DMSO. The plates were analyzed with the Varioskan Lux and the Skanit software (Thermo Fisher Scientific, Waltham, MA, USA) at 570 nm and 630 nm.

#### 3.3.6. Statistical Analysis

All statistical tests were performed using GraphPad Prism v.6.0c software. All viability data are shown as the mean ± standard error of the mean (SEM). Differences in the number of abnormal/fragmented eggs were analyzed by Student’s *t*-test. *P*-values < 0.05 was considered to be statistically significant.

## 4. Conclusions

1,2,4-Thiadiazole alkaloids are rarely found in natural products and, to our knowledge, polyaurine B represents the first example of a marine natural 3-(*N*-methyl-methylcarbamate) substituted 1,2,4-thiadiazole alkaloid. Its structure was elucidated at first by spectroscopic means and by comparing its spectral feature to those reported in the literature for the few reference compounds [5,12,13,14]. Further support to the proposed (5-(4-methoxyphenyl)-1,2,4-thiadiazol-3-yl)(methyl)carbamate structure was gained by the quantum mechanical calculation of its ^13^C NMR chemical shifts profile and application of DP4+ statistical analysis, a kind of approach which is becoming an important tool for structure elucidation of natural products. Definitely, polyaurines A and B are not cytotoxic on mammalian NIH-3T3 cells and on different stages of *S. mansoni*, although polyaurine A, interestingly, impairs egg production in vitro representing, therefore, a natural molecule with interesting biological properties. The egg phenotype could be further investigated by electron microscopy analyses and comparative proteomic studies in order to identify polyaurine A-targets.

## Figures and Tables

**Table 1:** ^1^H (700 MHz) and ^13^C NMR (125 MHz) spectroscopic data ^a^ of polyaurines A (**1**) and B (**2**) in CDCl_3_.

**Pos**	1 *^a^*		2 *^a^*	
	*δ*_C_	*δ*_H_ (mult, *J* in Hz)	*δ*_C_	*δ*_H_ (mult, *J* in Hz)
**1**	130.7	-	123.2	-
**2**	131.1	8.17 (d, *J* = 8.2 Hz)	128.9	7.88 (d, *J* = 7.9 Hz)
**3**	113.0	6.89 (d, *J* = 8.2 Hz)	114.7	6.96 (d, *J* = 7.9 Hz)
**4**	162.4	-	162.7	-
**5**	113.0	6.89 (d, *J* = 8.2 Hz)	114.7	6.96 (d, *J* = 7.9 Hz)
**6**	131.1	8.17 (d, *J* = 8.2 Hz)	128.9	7.88 (d, *J* = 7.9 Hz)
**7**	177.9	-	187.1	-
**8**	159.8	-	165.8	-
**9**	156.7	-	154.9	-
**10**	53.8	3.86 (s)	53.6	3.85 (s)
**11**	55.2	3.84 (s)	55.5	3.87 (s)
**12**	32.3	3.52 (s)	36.1	3.54 (s)
***-NH***	-	9.28 (br. s); 10.58 (br. s)	-	-

*^a^*^1^H NMR and ^13^C NMR shifts are referenced to CDCl_3_ (*δ*_H_ = 7.26 ppm and *δ*_C_ = 77.0 ppm). The proton and carbon resonances were assigned by HSQC and HMBC experiments.

**Table 2:** ^13^C calculated and experimental NMR chemical shifts for structure **2a**–2**f**. Chemical shift data here reported were produced using tetramethylsilane (TMS) as reference compound.

-	Structure 2a	Structure 2b	Structure 2c	Structure 2d	Structure 2e	Structure 2f	Experimental Carbon
**1**	127.7	130.2	128.3	131.0	125.2	128.4	123.2
**2**	133.8	137.2	132.5	130.0	131.1	130.2	128.9
**3**	111.7	110.2	112.4	112.5	112.2	112.3	114.5
**4**	166.9	165.6	165.2	165.0	165.2	164.6	162.7
**5**	111.5	110.5	112.0	112.1	112.1	112.2	114.5
**6**	132.9	133.8	131.5	136.0	136.3	136.7	128.9
**7**	192.6	176.3	169.7	161.0	154.8	158.0	187.2
**8**	170.8	185.5	163.7	158.7	155.0	157.6	165.9
**9**	157.4	152.3	158.5	158.2	157.9	156.0	155.0
**10**	53.2	53.8	54.1	53.4	53.3	53.7	53.6
**11**	54.5	54.1	54.2	54.1	54.2	54.1	55.5
**12**	35.7	34.7	32.3	35.7	36.4	38.4	36.2

**Table 3:** The calculation results of structures **2a**–**2f** with mean absolute errors (MAE) values and DP4+ probabilities.

-	MAE Value (ppm)		^13^C Data DP4+ Probability		
	^13^C MAE	^13^C CMAE	sDP4+	uDP4+	DP4+
**Structure 2a**	3.2	1.7	100.00%	100.00%	100.00%
**Structure 2b**	5.6	5.3	0.00%	0.00%	0.00%
**Structure 2c**	3.9	4.2	0.00%	0.00%	0.00%
**Structure 2d**	5.1	6.4	0.00%	0.00%	0.00%
**Structure 2e**	5.6	7.3	0.00%	0.00%	0.00%
**Structure 2f**	5.2	6.6	0.00%	0.00%	0.00%

## Supplementary Materials

The following are available online at https://www.mdpi.com/1660-3397/17/5/278/s1, Figure S1. HRESIMS spectrum of polyaurine A (**1**); Figure S2. ^1^H NMR spectrum of polyaurine A (**1**) in CDCl_3_; Figure S3. ^13^C NMR spectrum of polyaurine A (**1**) in CDCl_3_; Figure S4. HSQC spectrum of polyaurine A (**1**) in CDCl_3_; Figure S5. COSY spectrum of polyaurine A (**1**) in CDCl_3_; Figure S6. HMBC spectrum of polyaurine A (**1**) in CDCl_3_; Figure S7. HRESI-MS spectrum of polyaurine B (**2**); Figure S8. ^1^H NMR spectrum of polyaurine B (**2**) in CDCl_3_; Figure S9. ^13^C NMR spectrum of polyaurine B (**2**) in CDCl_3_; Figure S10. HSQC spectrum of polyaurine B (**2**) in CDCl_3_; Figure S11. COSY spectrum of polyaurine B (**2**) in CDCl_3_; Figure S12. HMBC spectrum of polyaurine B (**2**) in CDCl3; Figure S13. HRESI-MS spectrum of compound **3**; Figure S14. ^1^H NMR spectrum of compound **3** in CDCl_3_; Figure S15. HRESI-MS spectrum of compound **4**; Figure S16. ^1^H NMR spectrum of compound **4** in CDCl_3_; Figure S17. HRESI-MS spectrum of compound **5**; Figure S18. ^1^H NMR spectrum of compound **5** in CDCl_3_; Figure S19. HRESI-MS spectrum of compound **6**; Figure S20. ^1^H NMR spectrum of compound **6** in CDCl_3_; Figure S21. HRESI-MS spectrum of compound **7**; Figure S22. ^1^H NMR spectrum of compound **7** in CDCl_3_; Figure S23. HRESI-MS spectrum of compound **8**; Figure S24. ^1^H NMR spectrum of compound **8** in CDCl_3_.
