# Supplementary material for: Chemical Investigation of the Indonesian Tunicate Polycarpa aurata and Evaluation of the Effects Against Schistosoma mansoni of the Novel Alkaloids Polyaurines A and B
Source: Mar Drugs. 2019 May 10;17(5):278. doi: 10.3390/md17050278 (PMC6562961; doi:10.3390/md17050278)
Supplement: Supplementary file 1 [file marinedrugs-17-00278-s001.pdf]

Chemical Investigation of the Indonesian Tunicate *Polycarpa aurata* and Evaluation of the Effects Against *Schistosoma mansoni* of the Novel Alkaloids Polyaurines A and B.

Marcello Casertano[1], Concetta Imperatore[1], Paolo Luciano[1], Anna Aiello[1], Masteria Yunovilsa Putra[2], Roberto Gimmelli[3], Giovina Ruberti[3] and Marialuisa Menna[1,*]

[1] The NeaNat Group, Department of Pharmacy, University of Naples "Federico II", Via D. Montesano 49, 80131 Napoli, Italy; marcello.casertano@unina.it (M.C.); cimperat@unina.it (C.I.); pluciano@unina.it (P.L.); aiello@unina.it (A.A.)

[2] Research Center for Oceanography, Indonesian Institute of Sciences. Jl Pasir Putih Raya 1, DKI Jakarta 14430, Indonesia; mast001@lipi.go.id (M.Y.P.)

[3] Institute of Cell biology and Neurobiology, National Research Council, Campus A. Buzzati-Traverso, Via E. Ramarini, 32, 00015 Monterotondo (Roma), Italy; roberto.gimmelli@ibcn.cnr.it (R.G.); giovina.ruberti@cnr.it (G.R.)

* Correspondence: mlmenna@unina.it (M.M.); Tel.: +39-081-678518

TABLE OF CONTENTS

Figure S1. HRESIMS spectrum of polyaurine A (**1**)... 3

Figure S2. ^{1}H NMR spectrum of polyaurine A (**1**) in $CDCl_3$ (500 MHz).. 3

Figure S3. ^{13}C NMR spectrum of polyaurine A (**1**) in $CDCl_3$ (500 MHz)...4

Figure S4. HSQC spectrum of polyaurine A (**1**) in $CDCl_3$ (500 MHz)…….. 4

Figure S5. COSY spectrum of polyaurine A (**1**) in $CDCl_3$ (500 MHz)... 5

Figure S6. HMBC spectrum of polyaurine A (**1**) in $CDCl_3$ (500 MHz).. 5

Figure S7. HRESI-MS spectrum of polyaurine B (**2**).. 6

Figure S8. ^{1}H NMR spectrum of polyaurine B (**2**) in $CDCl_3$ (700 MHz).. 6

Figure S9. ^{13}C NMR spectrum of polyaurine B (**2**) in $CDCl_3$ (700 MHz).. 7

Figure S10. HSQC spectrum of polyaurine B (**2**) in $CDCl_3$ (700 MHz).. 7

Figure S11. COSY spectrum of polyaurine B (**2**) in $CDCl_3$ (700 MHz).. 8

Figure S12. HMBC spectrum of polyaurine B (**2**) in $CDCl_3$ (700 MHz).. 8

Figure S13. HRESI-MS spectrum of compound **3**.. 9

Figure S14. ^{1}H NMR spectrum of compound **3** in $CDCl_3$ (700 MHz)... 9

Figure S15. COSY spectrum of compound **3** in $CDCl_3$ (700 MHz).. 10

Figure S16. HRESI-MS spectrum of compound **4** ...…... 10

Figure S17. ^{1}H NMR spectrum of compound **4** in $CDCl_3$ (700 MHz)... 11

Figure S18. HSQC spectrum of compound **4** in $CDCl_3$ (700 MHz)... 11

Figure S19. COSY spectrum of compound **4** in $CDCl_3$ (700 MHz).. 12

Figure S20. HMBC spectrum of compound **4** in $CDCl_3$ (700 MHz).. 12

Figure S21. HRESI-MS spectrum of compound **5** …….. 13

Figure S22. ^{1}H NMR spectrum of compound **5** in $CDCl_3$ (700 MHz)... 13

Figure S23. HRESI-MS spectrum of compound **6** ...…... 14

Figure S24. ^{1}H NMR spectrum of compound **6** in $CDCl_3$ (500 MHz).. 14

Figure S25. HRESI-MS spectrum of compound **7** ...…... 15

Figure S26. ^{1}H NMR spectrum of compound **7** in $CDCl_3$ (500 MHz).. 15

Figure S27. HRESI-MS spectrum of compound **8** ……….. 16

Figure S28. ^{1}H NMR spectrum of compound **8** in $CDCl_3$ (700 MHz).. 16

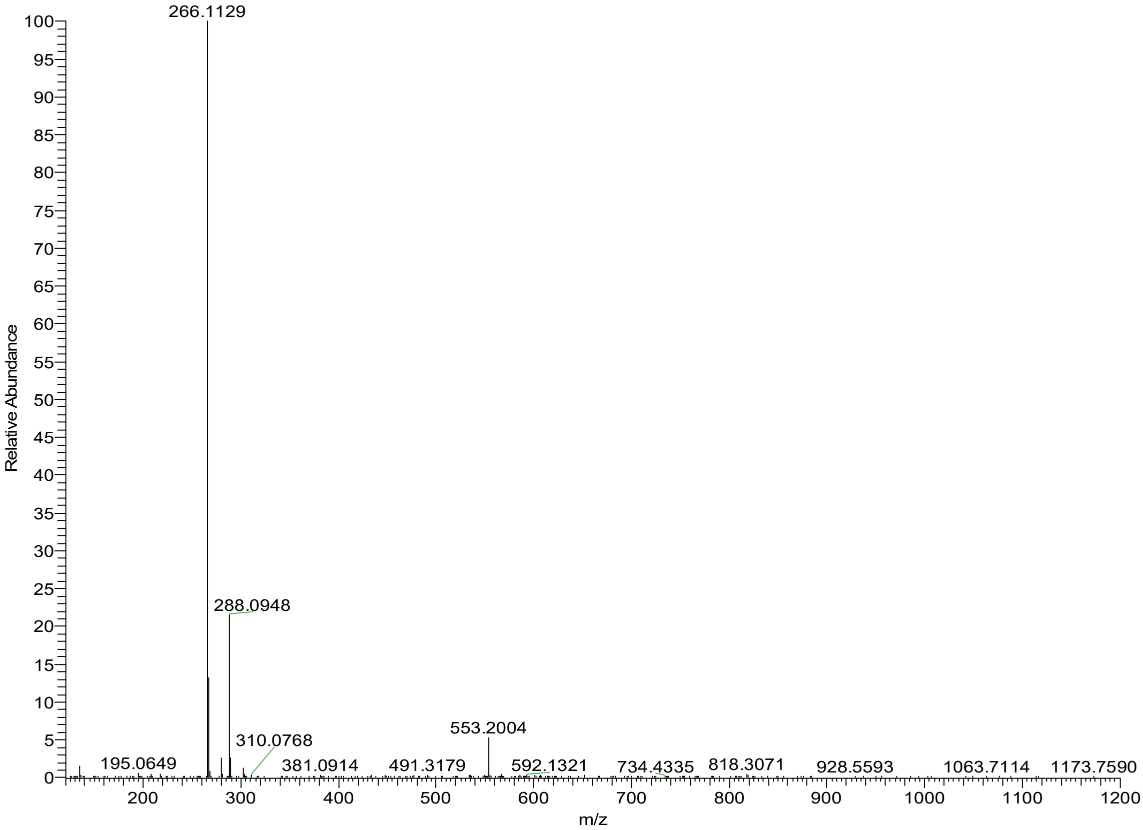

Figure S1. HRESI-MS spectrum of polyaurine A (**1**)

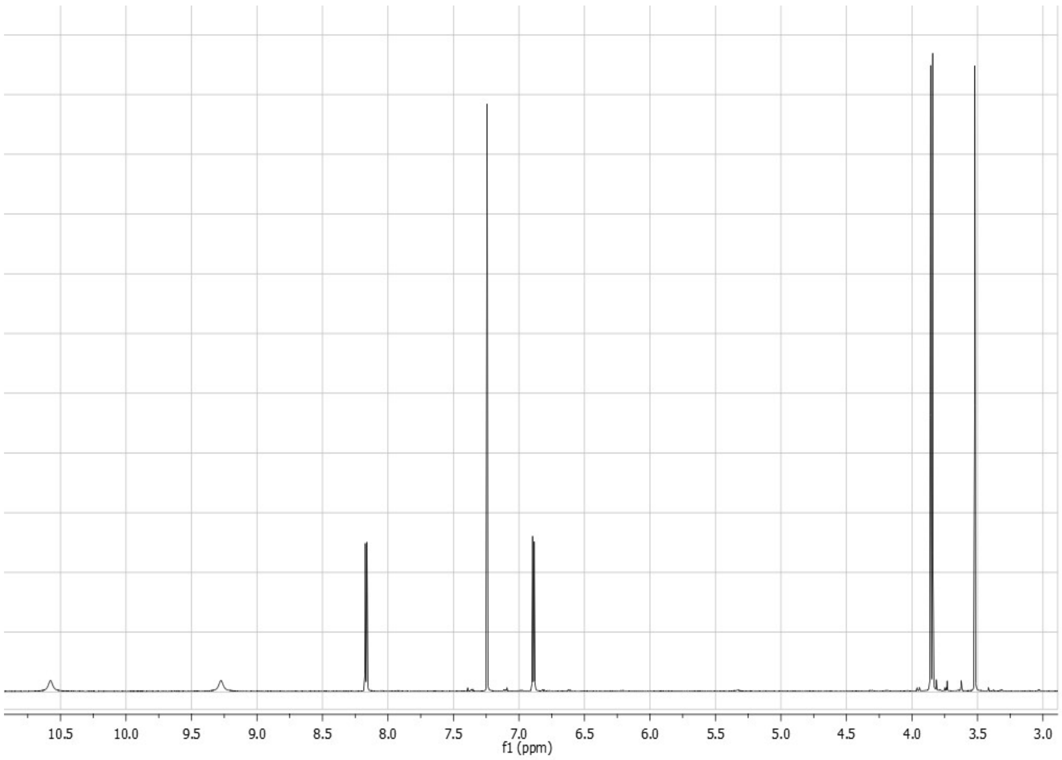

Figure S2. ^{1}H NMR spectrumof polyaurine A (**1**) in $CDCl_3$ (500 MHz)

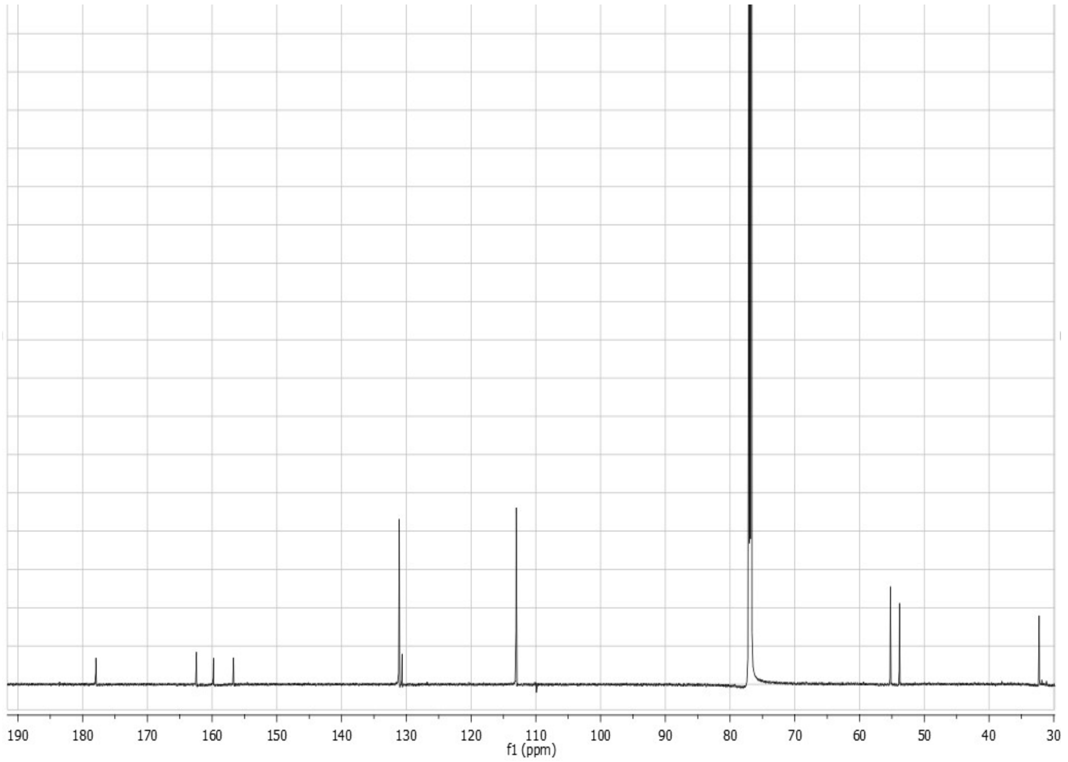

Figure S3. ^{13}C NMR spectrum of polyaurine A (**1**) in $CDCl_3$ (500 MHz)

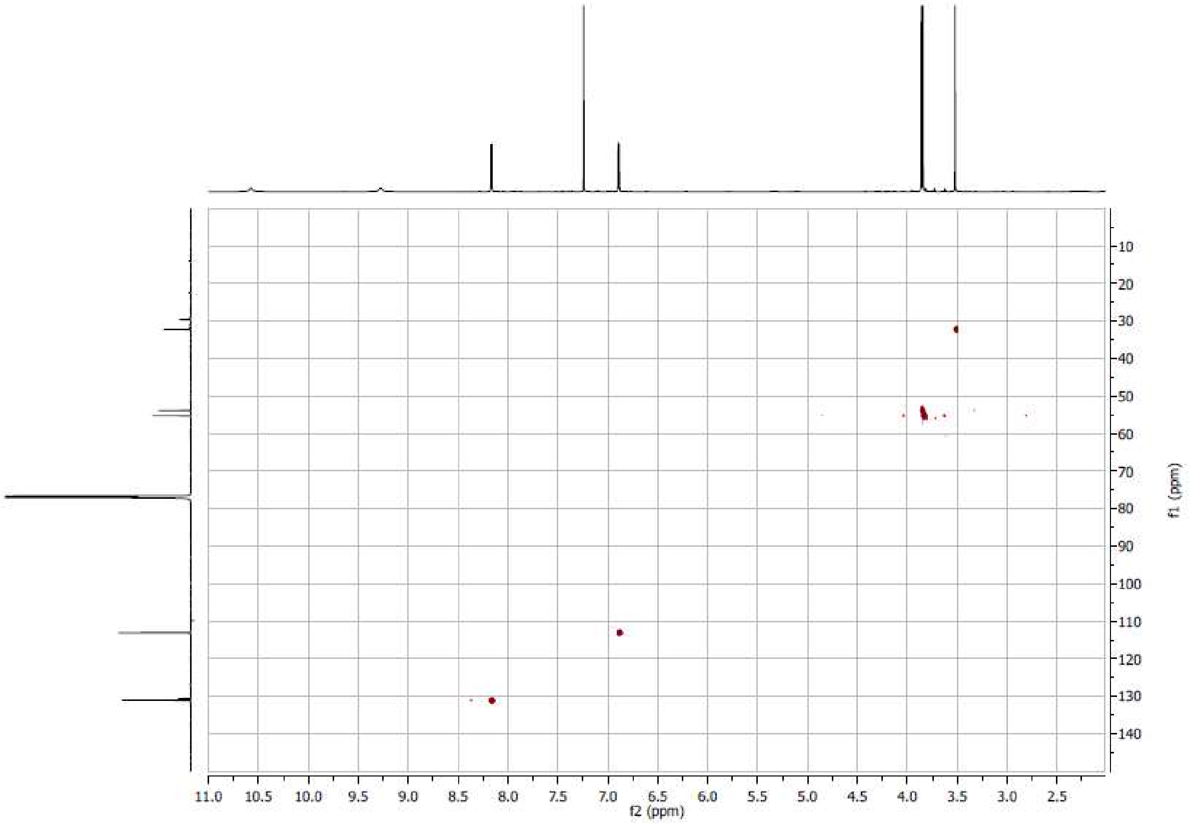

Figure S4. HSQC spectrum of polyaurine A (**1**) in $CDCl_3$ (500 MHz)

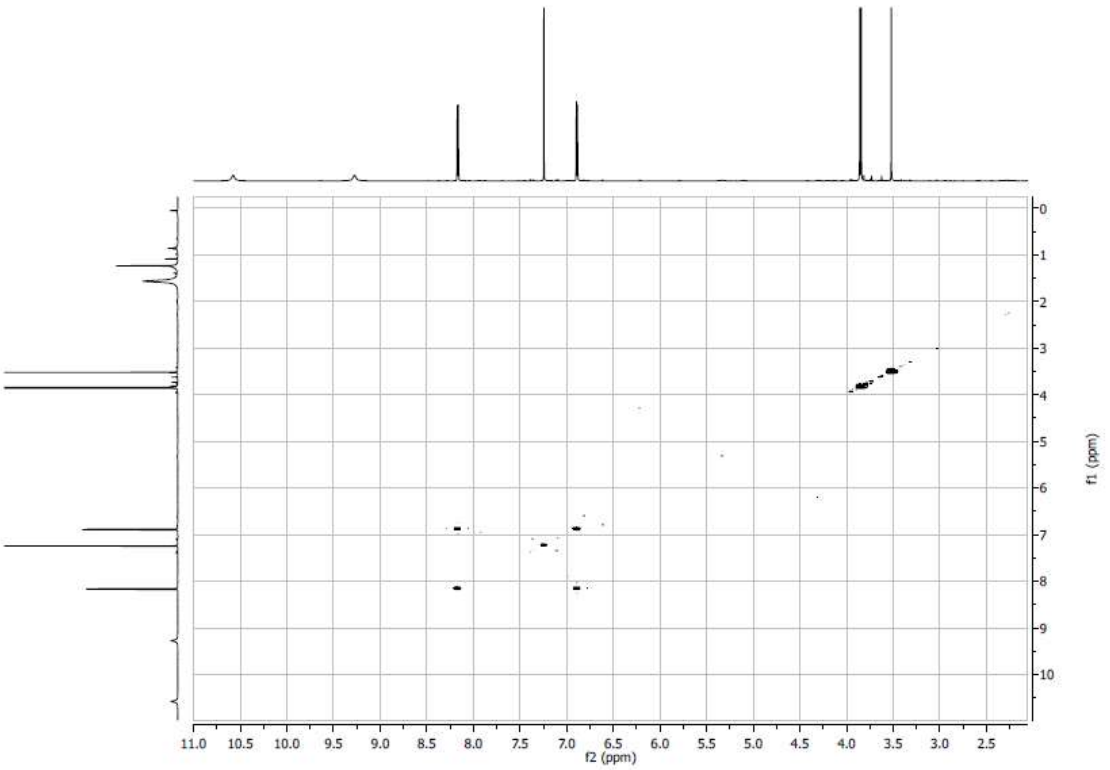

Figure S5. COSY spectrum of polyaurine A (**1**) in $CDCl_3$ (500 MHz)

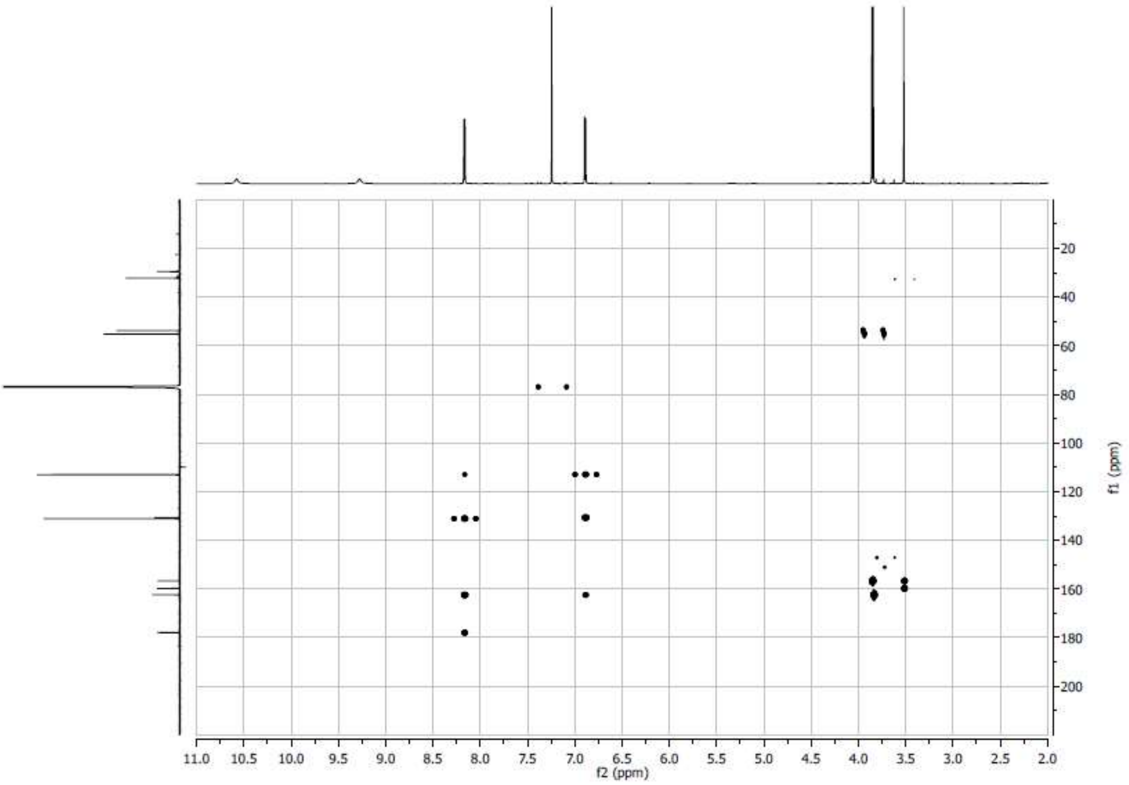

Figure S6. HMBC spectrum of polyaurine A (**1**) in $CDCl_3$ (500 MHz)

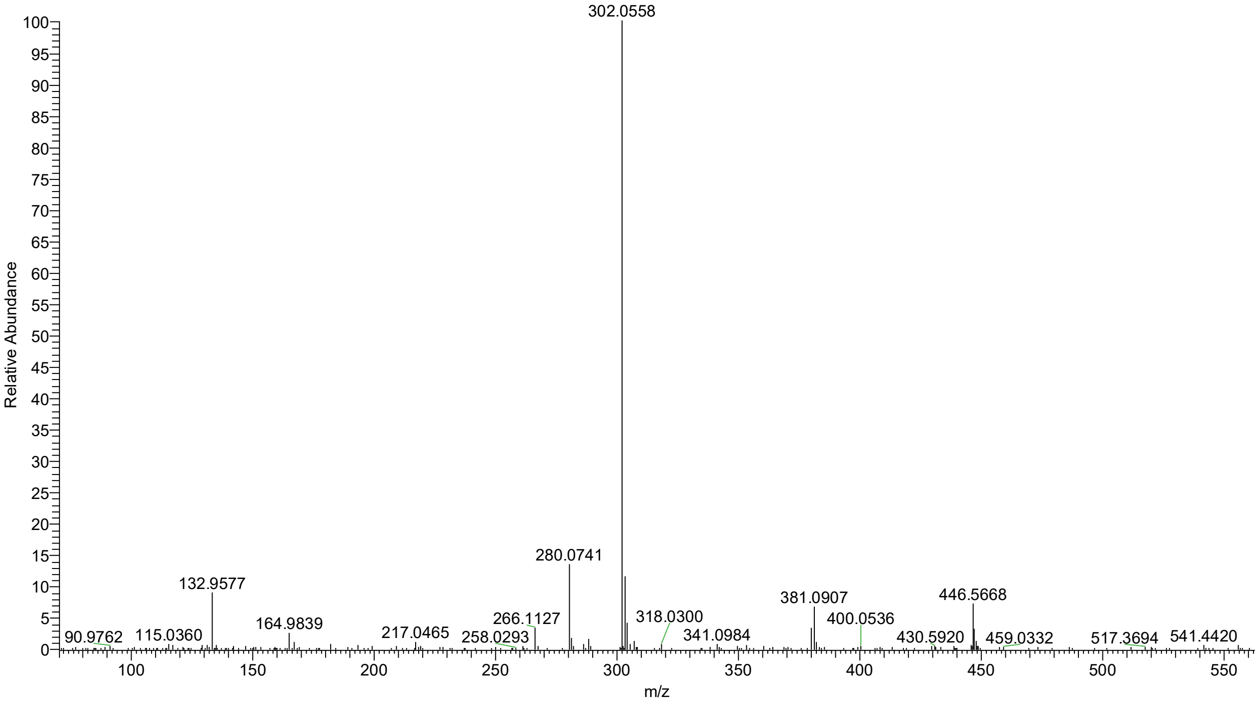

Figure S7. HRESI-MS spectrum of polyaurine B (**2**)

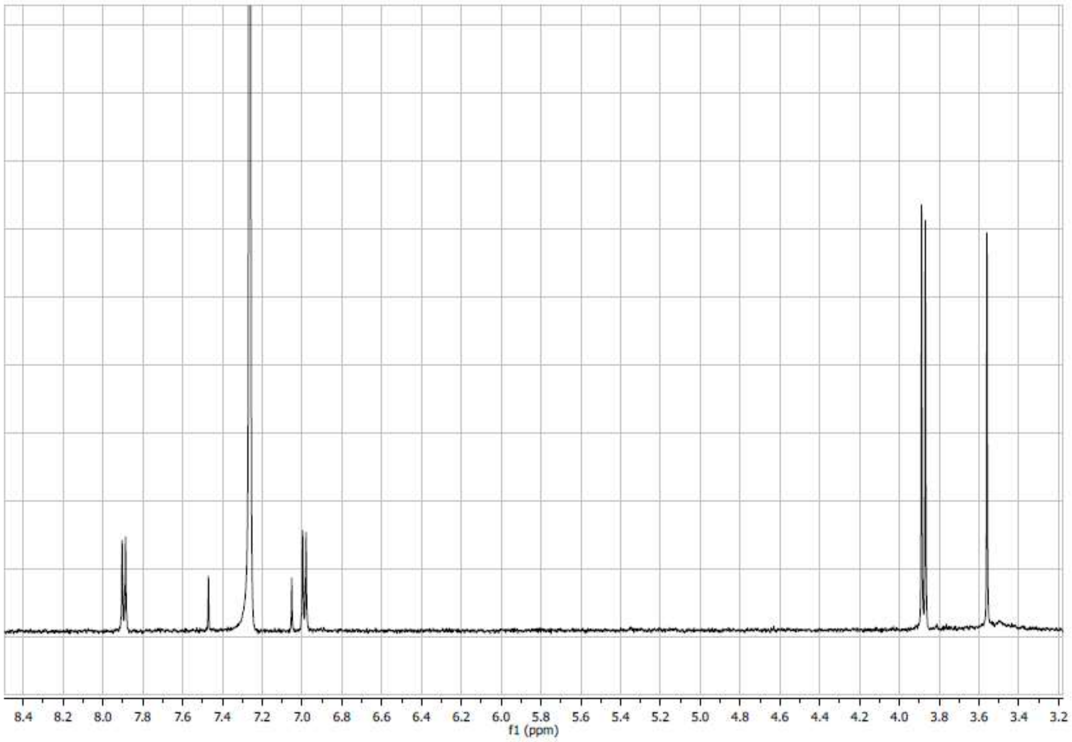

Figure S8. ^{1}H NMR spectrum of polyaurine B (**2**) in $CDCl_3$ (700 MHz)

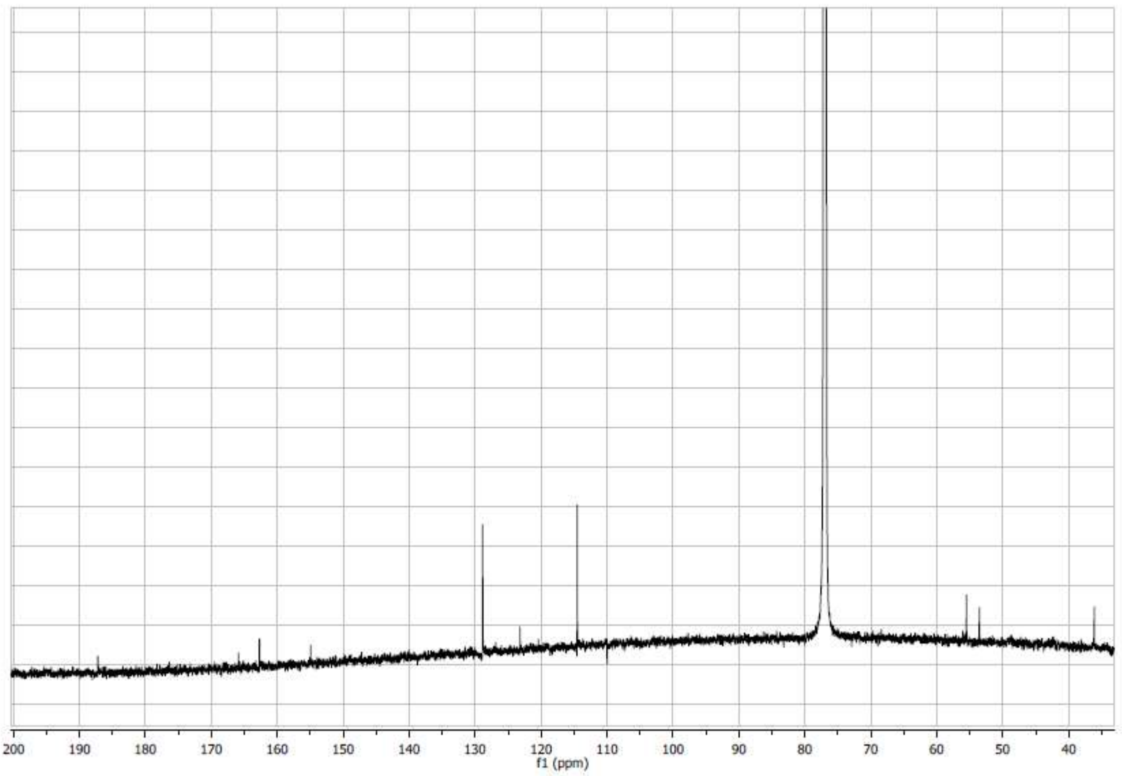

Figure S9.^{13}C NMR spectrum of polyaurine B (**2**) in $CDCl_3$ (700 MHz)

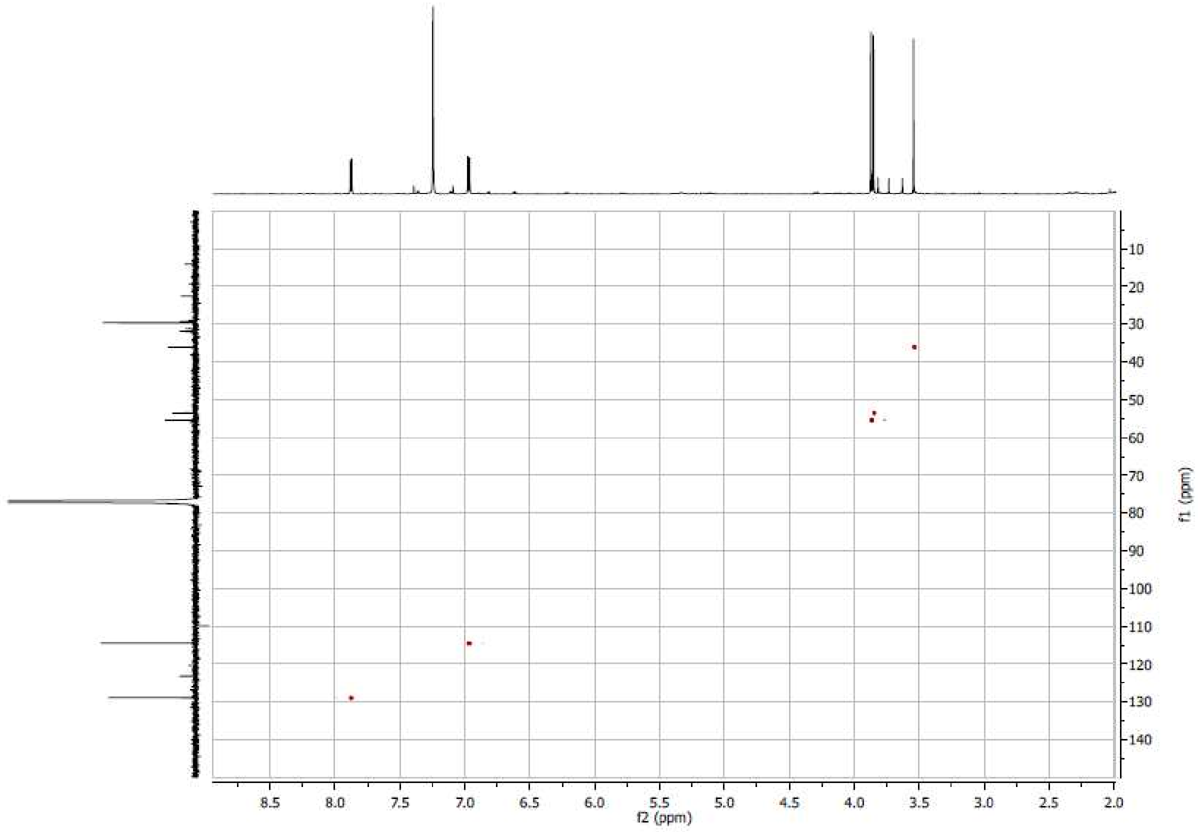

Figure S10. HSQC spectrum of polyaurine B (**2**) in $CDCl_3$ (700 MHz)

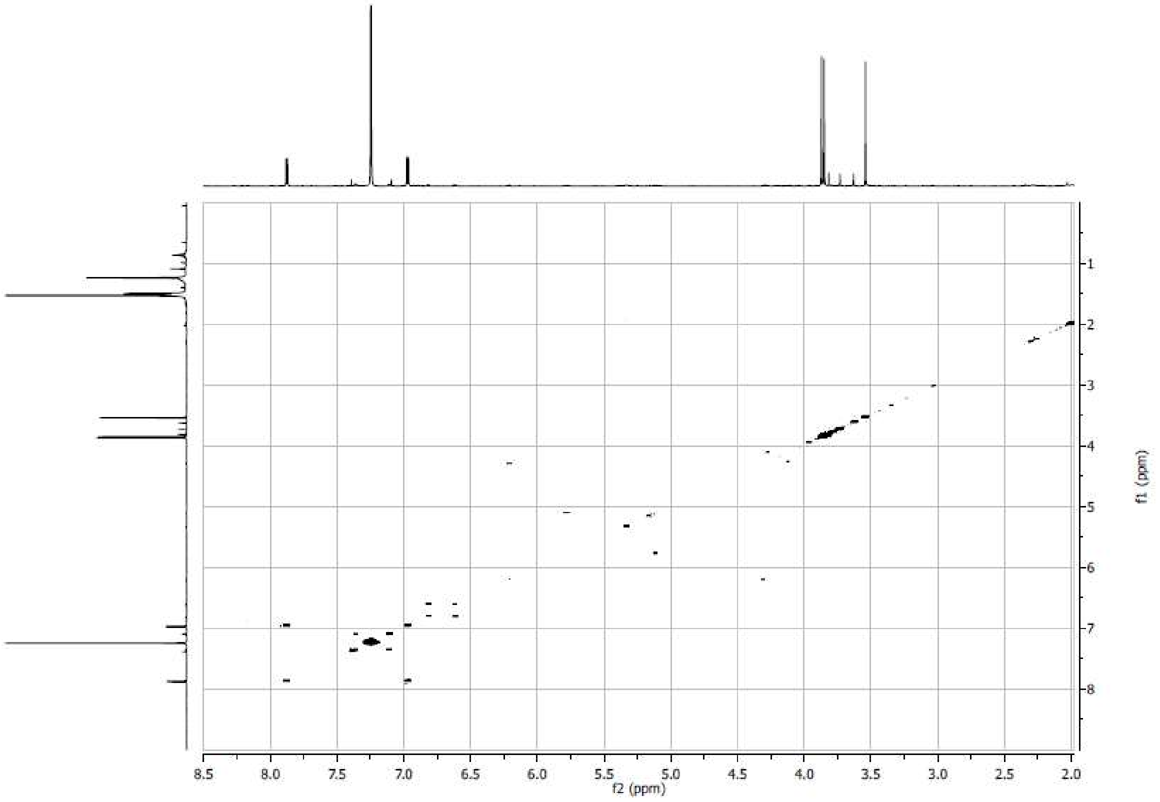

Figure S11. COSY spectrum of polyaurine B (**2**) in $CDCl_3$ (700 MHz)

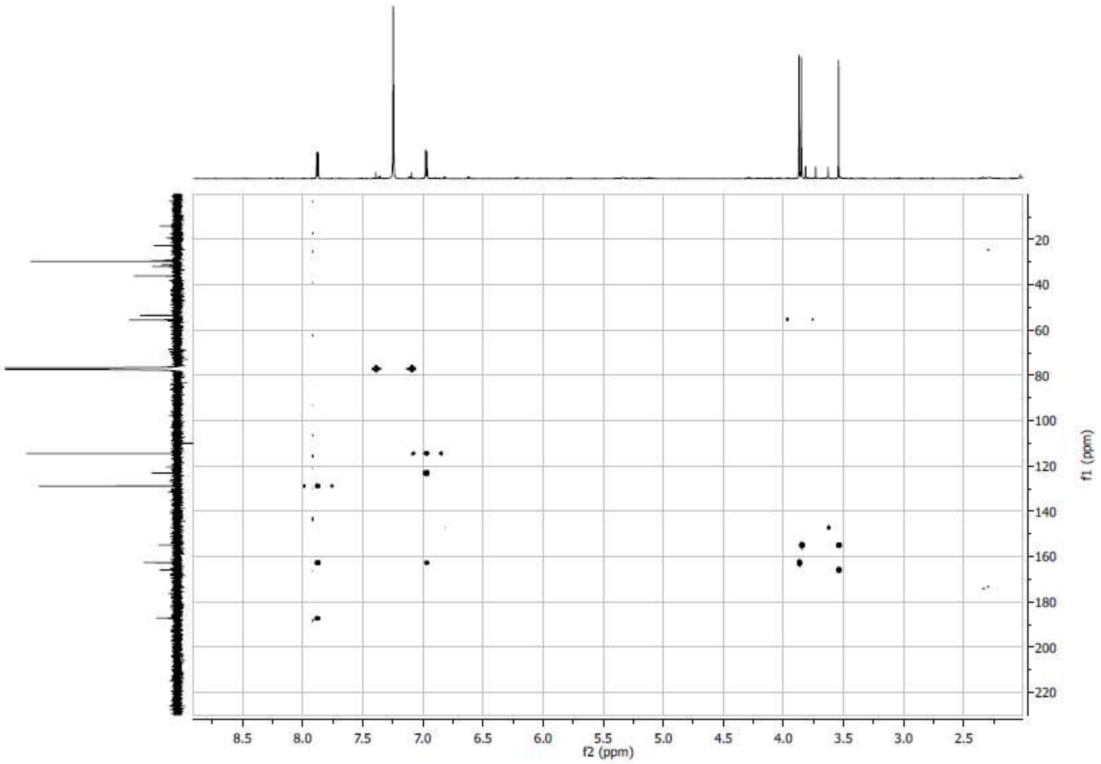

Figure S12. HMBC spectrum of polyaurine B (**2**) in $CDCl_3$ (700 MHz)

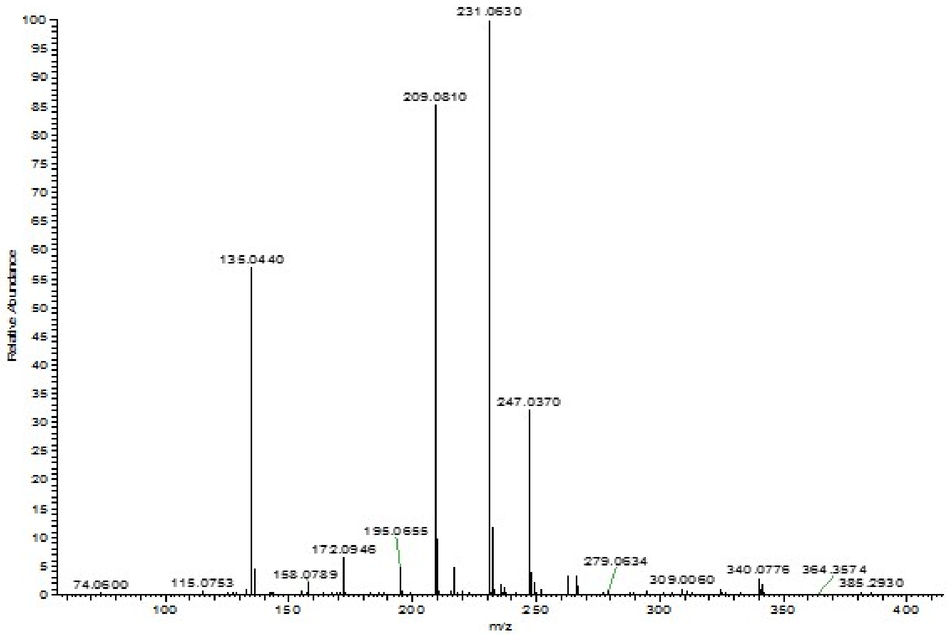

Figure S13. HRESI-MS spectrum of compound **3**

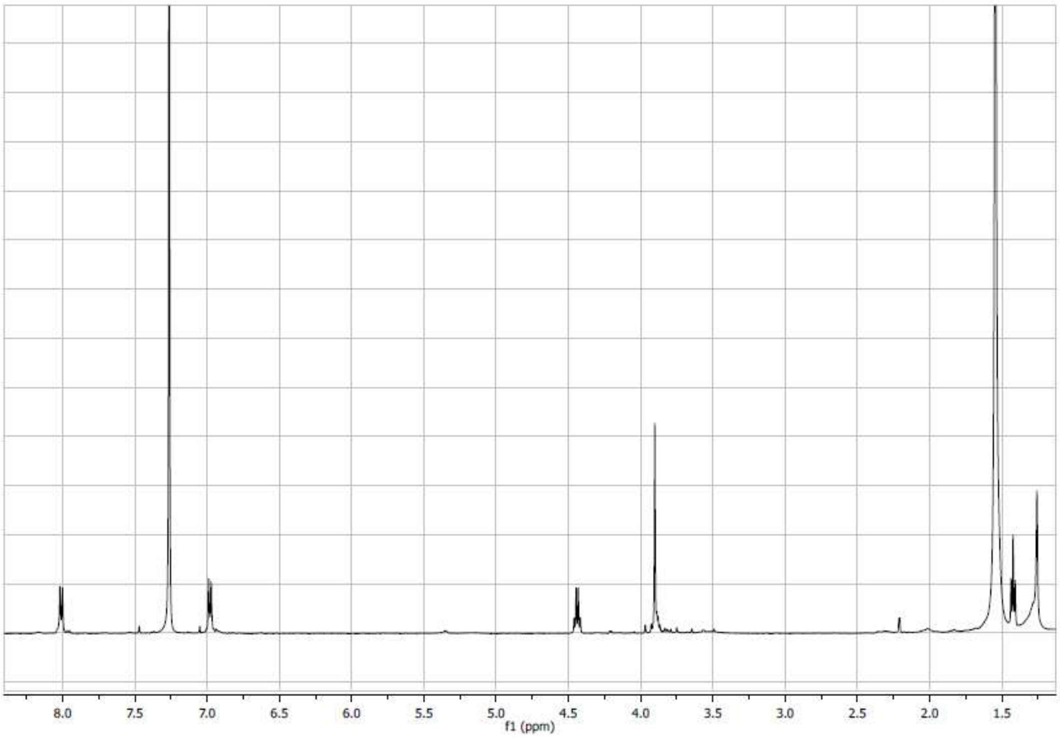

Figure S14. ^{1}H NMR spectrum of compound **3** in $CDCl_3$ (700 MHz)

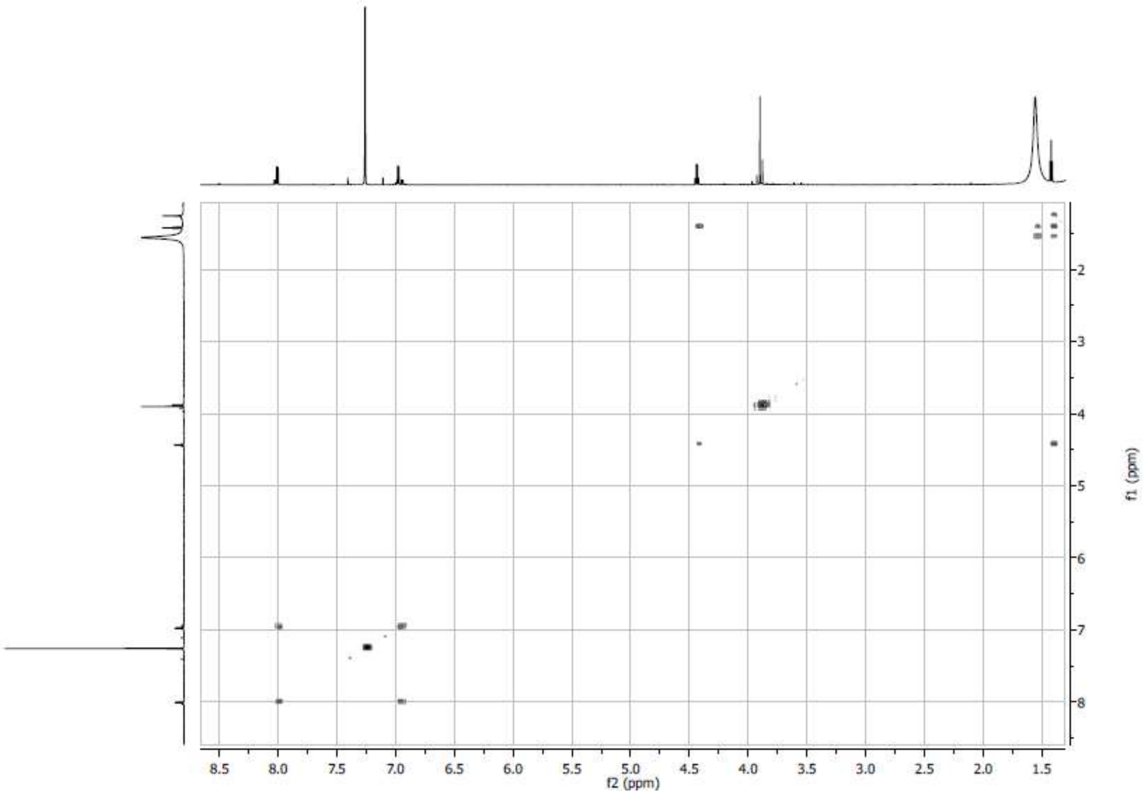

Figure S15. COSY spectrum of compound **3** in $CDCl_3$ (700 MHz)

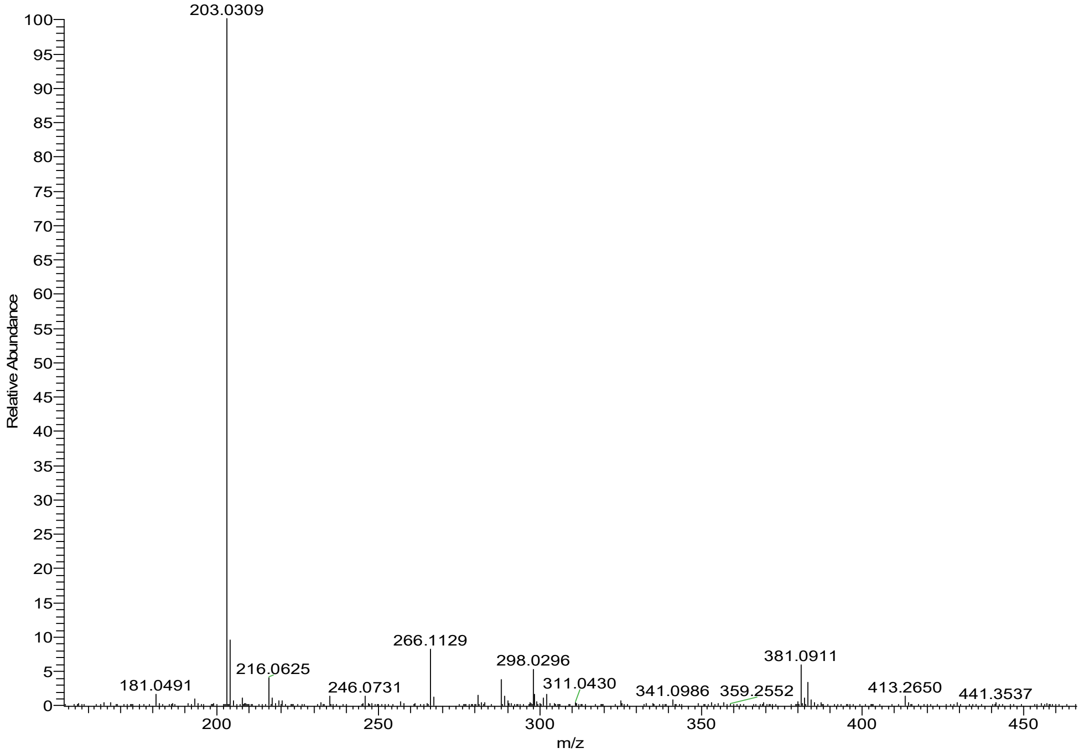

Figure S16. HRESI-MS spectrum of compound **4**

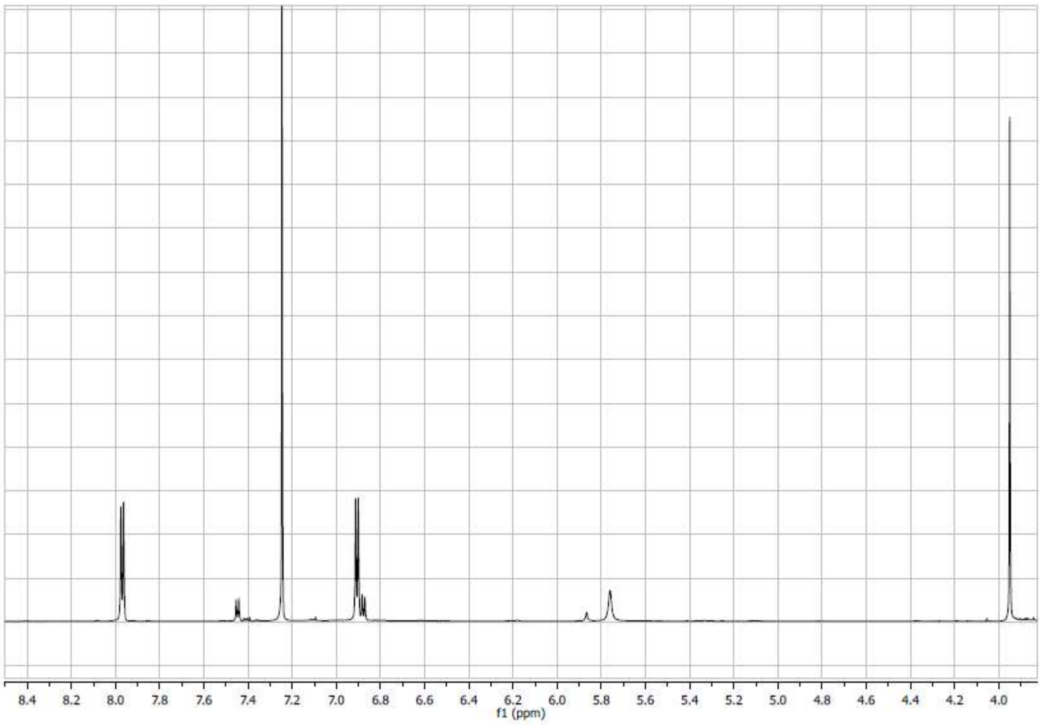

Figure S17. ^{1}H NMR spectrum of compound **4** in $CDCl_3$ (700 MHz)

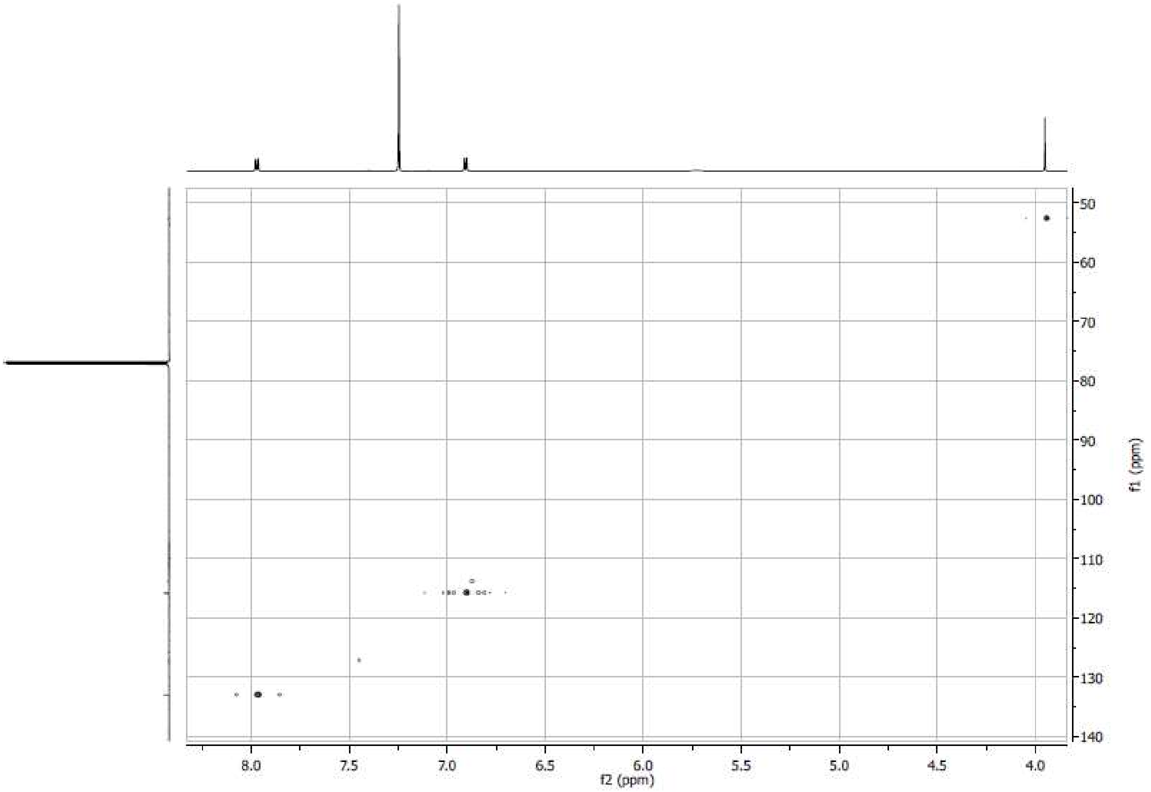

Figure S18. HSQC spectrum of compound **4** in $CDCl_3$ (700 MHz)

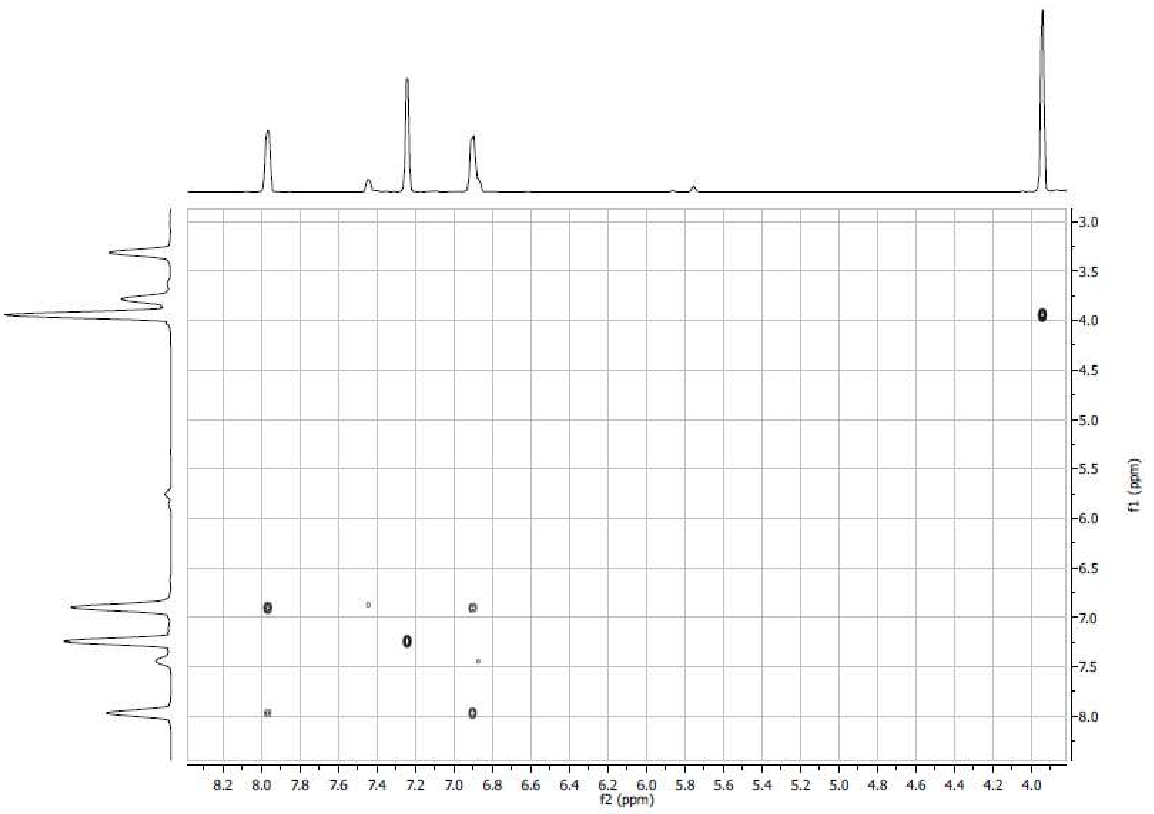

Figure S19. COSY spectrum of compound **4** in $CDCl_3$ (700 MHz)

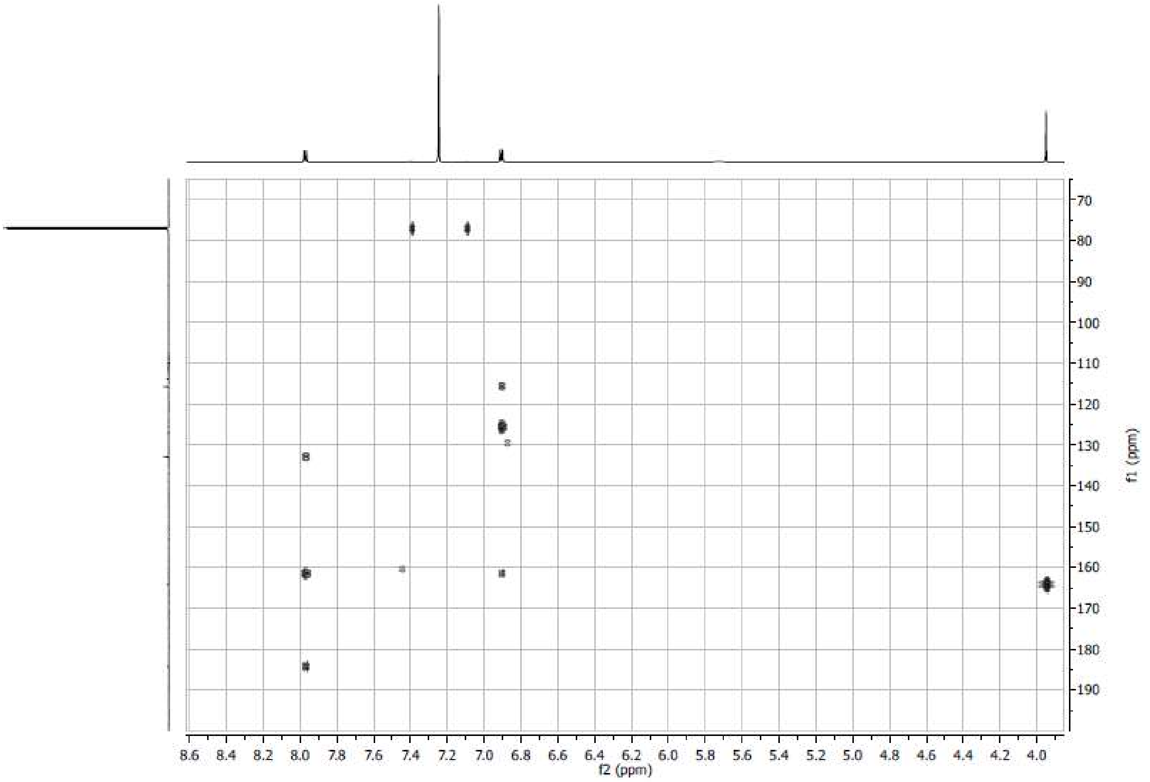

Figure S20. HMBC spectrum of compound **4** in $CDCl_3$ (700 MHz)

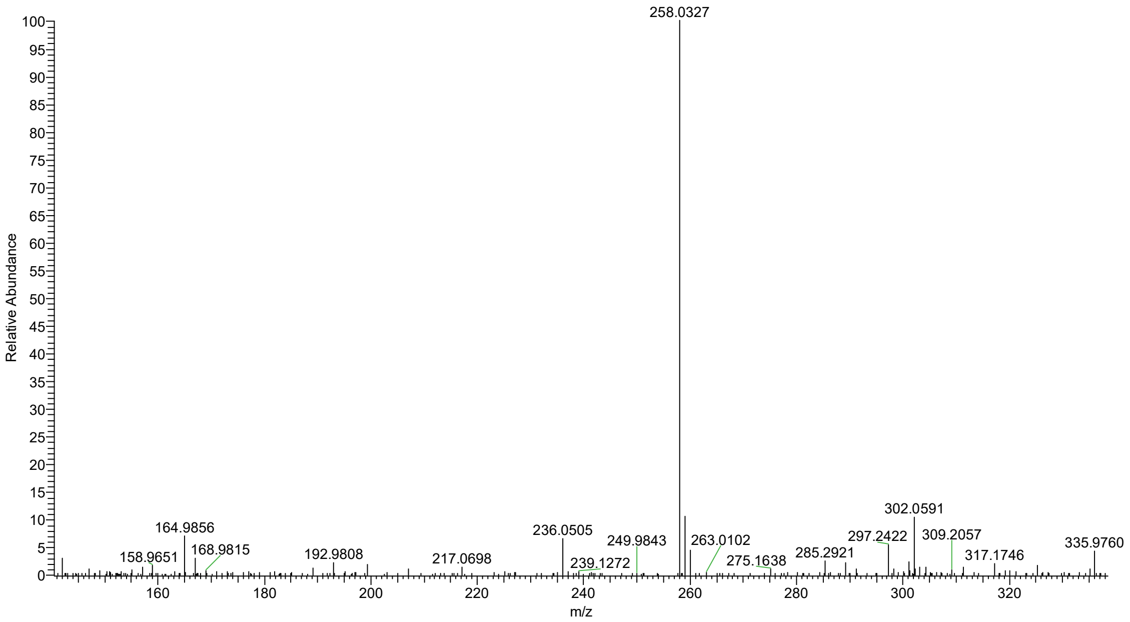

Figure S21. HRESI-MS spectrum of compound **5**

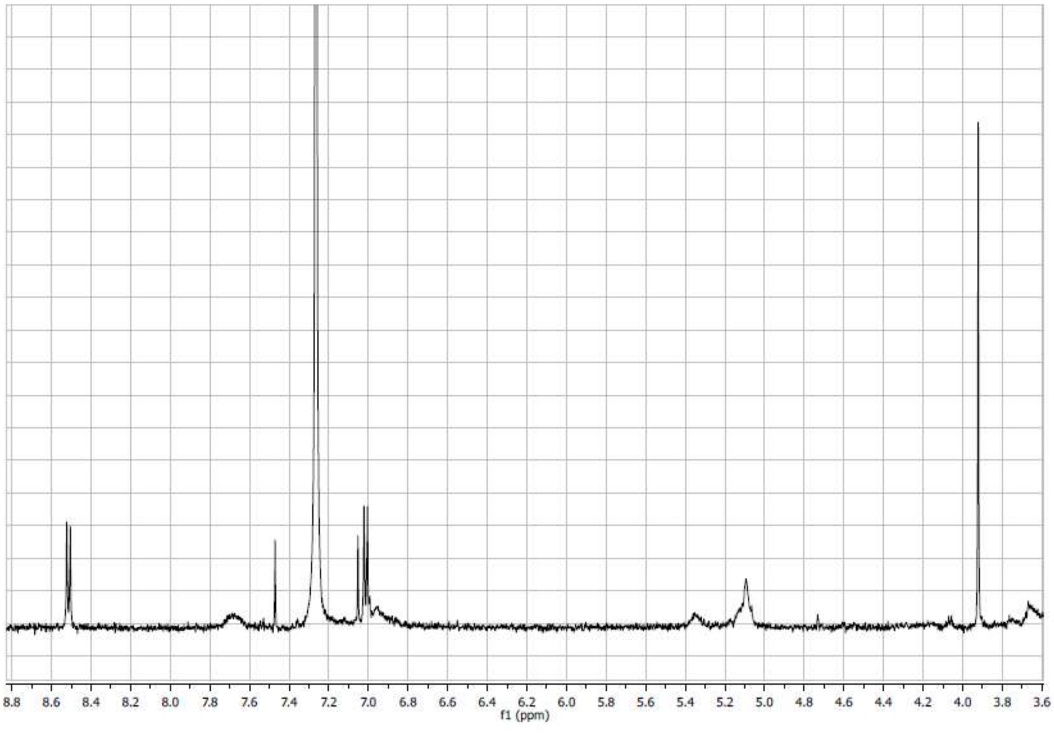

Figure S22. ^{1}H NMR spectrum of compound **5** in $CDCl_3$ (700 MHz)

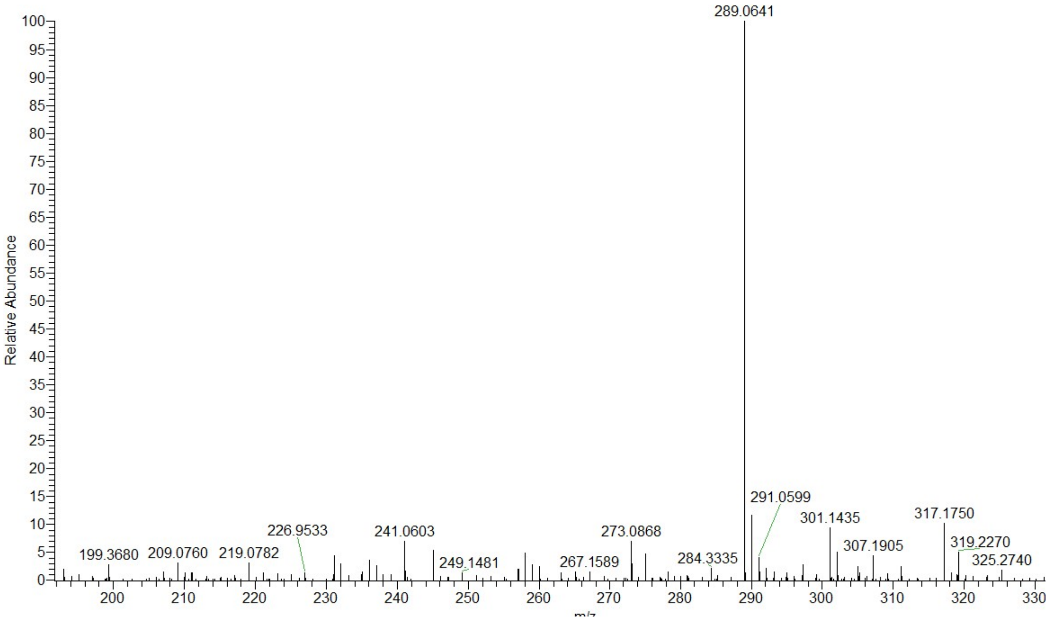

Figure S23. HRESI-MS spectrum of compound **6**

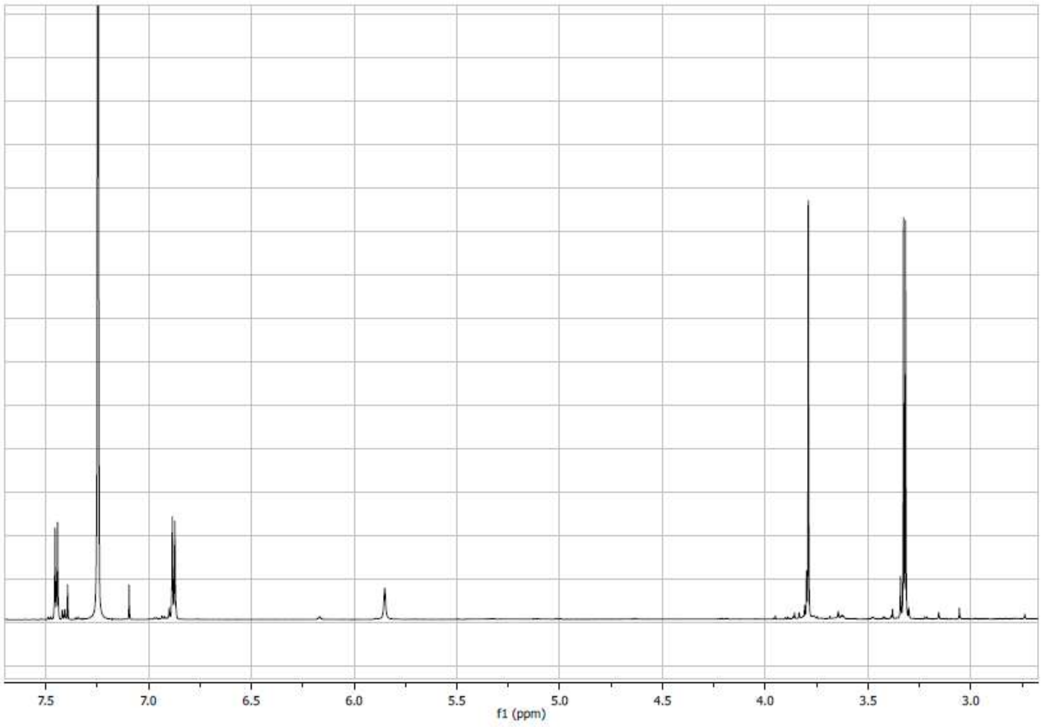

Figure S24. ^{1}H NMR spectrum of compound **6** in $CDCl_3$ (500 MHz)

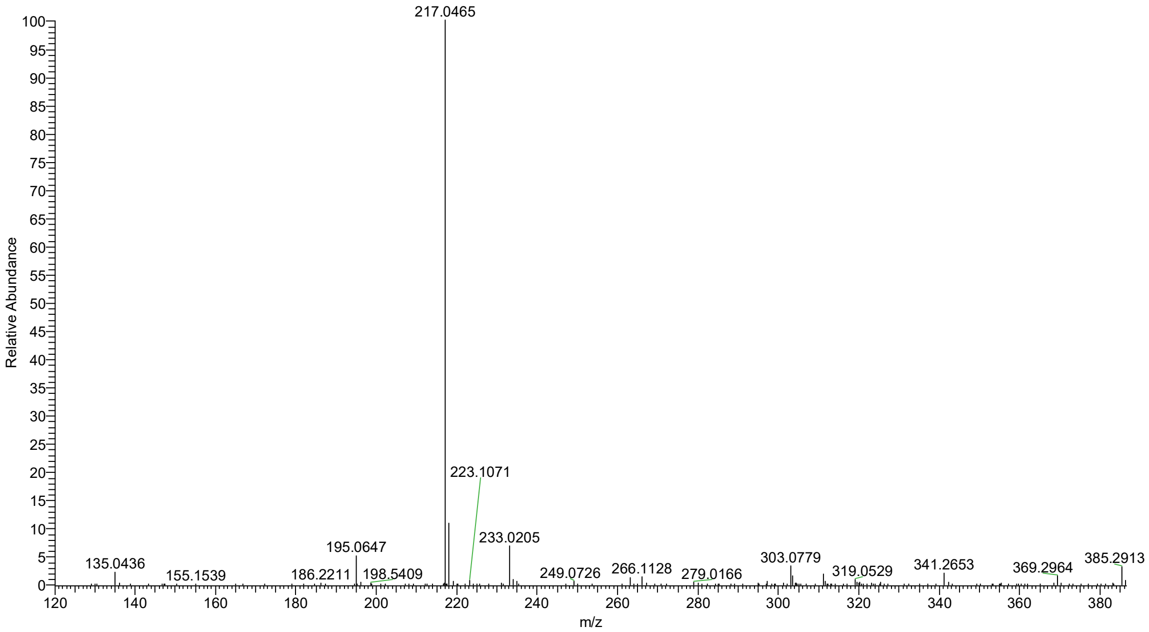

Figure S25. HRESI-MS spectrum of compound **7**

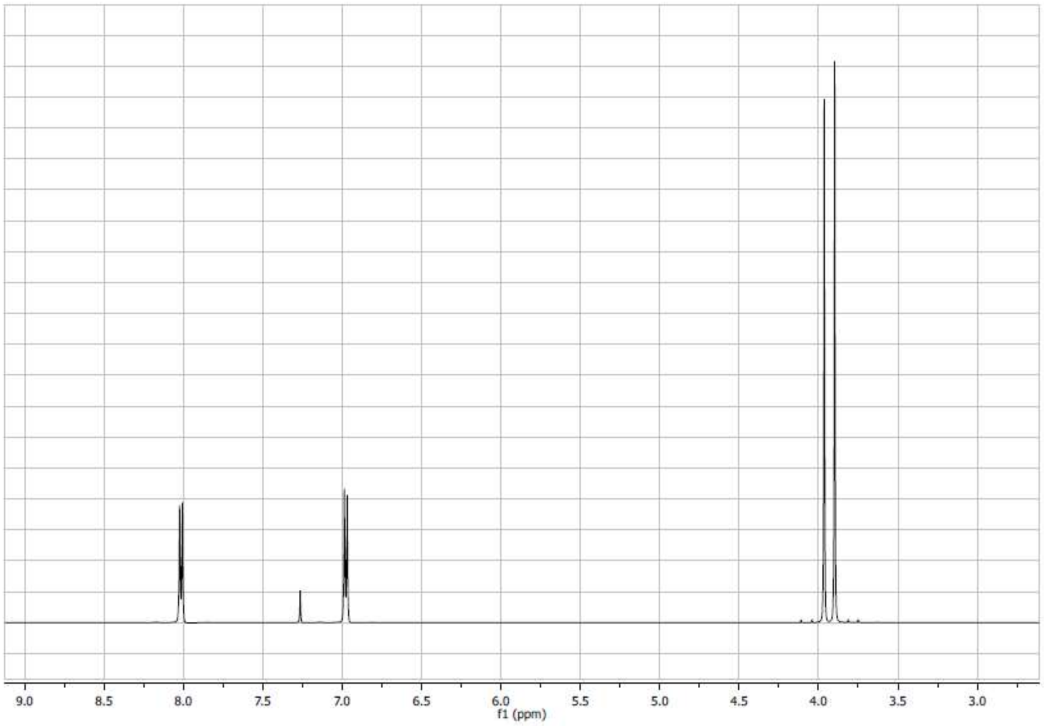

Figure S26. ^1H NMR spectrum of compound **7** in $CDCl_3$ (500 MHz)

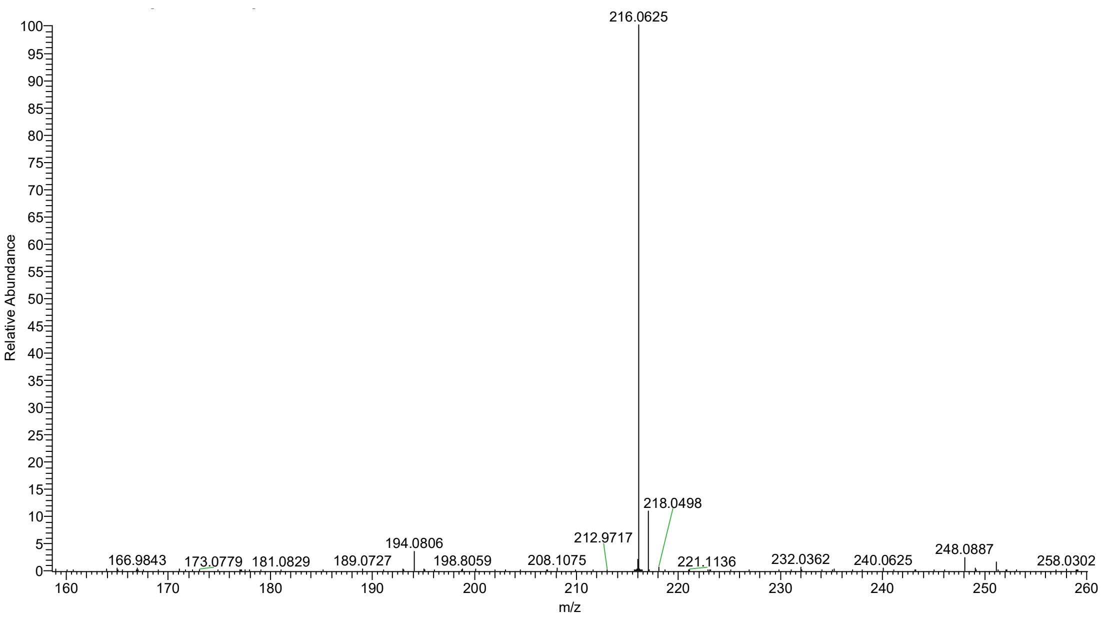

Figure S27. HRESI-MS spectrum of compound **8**

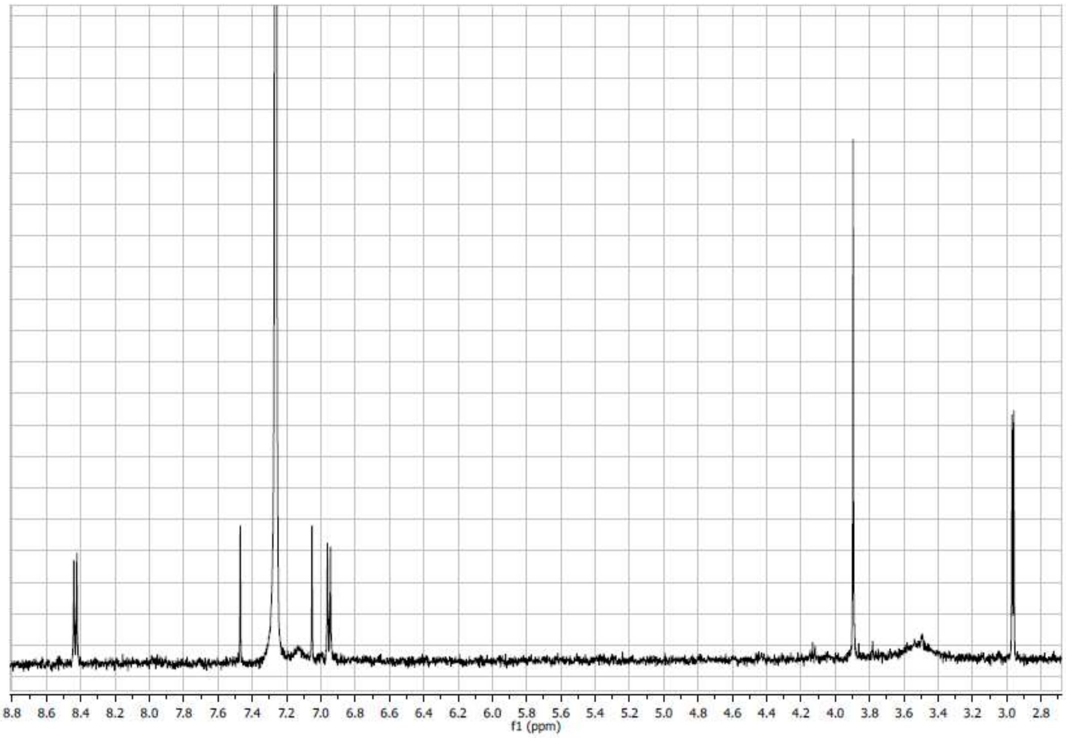

Figure S28. ^{1}H NMR spectrum of compound **8** in $CDCl_3$ (700 MHz)
